# Deciphering the dialogue between the bovine blastocyst and the uterus: embryo-induced alterations in extracellular vesicle protein content from an ex vivo model and the in vivo environment

**DOI:** 10.1186/s40104-025-01270-1

**Published:** 2025-10-24

**Authors:** Rosane Mazzarella, José María Sánchez, Sandra Guisado Egido, Michael McDonald, Alberto Álvarez-Barrientos, Esperanza González, Juan Manuel Falcón-Pérez, Mikel Azkargorta, Félix Elortza, Maria Encina González, Pat Lonergan, Dimitrios Rizos, Beatriz Fernandez-Fuertes

**Affiliations:** 1 Department of Animal Reproduction, INIA-CSIC, Madrid, Spain; 2 Department of Anatomy and Embryology, Veterinary Faculty, Complutense University of Madrid (UCM), Madrid, Spain; 3 School of Agriculture and Food Science, University College Dublin, Belfield, Dublin, Ireland; 4 Servicio de Técnicas Aplicadas a La Biociencia, Universidad de Extremadura, Badajoz, Spain; 5 Exosomes Laboratory, Center for Cooperative Research in Biosciences (CIC bioGUNE), Basque Research and Technology Alliance (BRTA), Derio, Spain; 6 IKERBASQUE, Basque Foundation for Science, Bilbao, Spain; 7 Centro de Investigación Biomédica en Red en El Área Temática de Enfermedades Hepáticas (CIBEReh), Madrid, Spain; 8 Proteomics Platform, Center for Cooperative Research in Biosciences (CIC bioGUNE), Basque Research and Technology Alliance (BRTA), Derio, Spain

**Keywords:** Bovine blastocysts, Early pregnancy, Embryonic extracellular vesicles, Embryo-maternal communication, Endometrial explants, Proteomics, Uterine fluid

## Abstract

**Backgroud:**

Efficient communication between the embryo and the endometrium is essential for the successful establishment and maintenance of pregnancy. Uterine-derived extracellular vesicles (EVs) contribute to embryo-maternal communication, supporting early embryonic development. This study aimed to: (i) compare the protein cargo of uterine fluid EVs (UF-EVs) from CYCLIC and PREGNANT heifers; (ii) characterize the protein profile of conditioned medium (CM)-EVs from endometrial explants cultured alone (EXPL) or co-cultured with five d 7 blastocysts (EXPL + EMB) in vitro; and (iii) compare the EV protein cargo between the in vivo and in vitro models (i.e., EXPL vs. CYCLIC and EXPL + EMB vs. PREGNANT).

**Results:**

We identified 1,459 and 1,752 proteins in the UF-EVs of CYCLIC and PREGNANT heifers, respectively. Among these, 12 were exclusive to CYCLIC, and 18 were exclusive to PREGNANT. Among the 1,329 proteins identified in both groups, 16 were differently abundant; ten were more abundant, and six were less abundant in UF-EVs from PREGNANT heifers. In vivo, the changes in UF-EV protein cargo induced by the presence of a blastocyst were related to inflammatory and immune responses, endometrial receptivity, and support of early embryonic development by promoting cell polarity, cell–cell adhesion, and stem cell differentiation. In vitro, we identified 1,501 proteins in the CM-EVs from EXPL, 1,975 in the CM-EVs from EXPL + EMB, and 82 in the CM-EVs from EMB. Additionally, 50 proteins were unique to EXPL + EMB, and another 33 were differentially abundant due to the synergistic interaction between the embryo and the endometrium. These proteins are involved in embryonic development, regulation of stem cell differentiation, establishment and maintenance of cell polarity, interferon tau (IFNT)-mediated cell signaling, endometrial receptivity, and immune modulation. Although there are qualitative and quantitative differences between in vivo and in vitro-derived EVs, UF-EVs from CYCLIC heifers compared to CM-EVs from EXPL, as well as UF-EVs from PREGNANT heifers compared to CM-EVs from EXPL + EMB shared common proteins.

**Conclusions:**

These findings highlight the pivotal role of EVs in embryo-maternal communication, suggesting that their protein cargo may actively contribute to the modulation of the uterine environment to support early embryonic development. Understanding these molecular interactions could provide valuable insights into the mechanisms of implantation and pregnancy establishment.

**Supplementary Information:**

The online version contains supplementary material available at 10.1186/s40104-025-01270-1.

## Introduction

Establishing and maintaining pregnancy requires reciprocal molecular communication between the embryo and the maternal reproductive tract. Despite the advances in assisted reproductive technologies that have made it possible to produce embryos in vitro to the stage of hatching blastocyst, the quality of the resulting embryos is inferior to those derived in vivo [1]. Specifically, embryos generated in vitro exhibit significant alterations in gene expression [2], metabolic changes [3], increased lipid accumulation [4], reduced tolerance to cryopreservation [5], and lower pregnancy success rates [6] when compared to their in vivo counterparts. Furthermore, pregnancy losses in cattle are most common in the pre-implantation window [7]. In both cases, the reduced quality of in vitro-produced embryos and these early pregnancy losses can be attributed to dysfunctions in the interaction between the embryo and the female reproductive tract, as well as the lack of an ideal physiological environment. These findings underscore the critical importance of embryo-maternal communication in the uterus, which regulates both endometrial and embryonic functions to ensure a successful pregnancy. 

Although embryo-endometrial interaction is most significant during maternal recognition of pregnancy [8], the early embryo can already modulate gene expression and the proteome of the endometrium, initiating an early embryo-maternal dialogue. Sponchiado et al. [9] demonstrated that physical proximity with the embryo facilitates paracrine regulation of endometrial function, as evidenced by changes in endometrial gene expression in response to the pre-hatching embryo in vivo. Additionally, changes in the metabolite composition of the UF are also observed in response to the pre-hatching bovine embryo [10]. In vitro, d 5 to 9 embryos can induce an anti-inflammatory response in both bovine endometrial epithelial cells (BEEC) and immune cells [11]. Moreover, d 8 embryos have been shown to induce expression changes in interferon-stimulated genes (ISGs) in endometrial explants [12].

Pre-implantation embryos produce a variety of biochemical signals, collectively termed embryotropins, which facilitate communication with the endometrium [13]. Recently, extracellular vesicles (EVs) have emerged as important mediators of maternal-embryonic signaling and as having a role in supporting early embryonic development. EVs are lipid bilayer-delimited nanoparticles actively released by cells into the extracellular space [14]. EVs modulate recipient cell functions and promote intercellular communication by delivering their bioactive cargo, including proteins, lipids, mRNAs, and microRNAs (miRNAs) [15]. Multiple studies have shown that embryos produce and release EVs that can be internalized by endometrial cells [16–18]. Recent findings indicate that EVs secreted by bovine embryos during blastocyst formation (d 5–7) can induce transcriptomic modifications in endometrial cells, particularly by activating interferon tau (IFNT) signaling [19]. Therefore, embryo-derived EVs may play an important role in facilitating embryo-maternal interactions during the pre-implantation period.

Extracellular vesicles are also present in the uterine fluid (UF), also known as histotroph, contributing to the maternal side of this reciprocal communication. Before implantation, the growth and development of the free-floating embryo are supported by the UF [18]. Produced by endometrial glandular cells, the UF also contains a critical mixture of biochemical components, including carbohydrates, lipids, amino acids, and growth factors [19]. UF-EVs have been identified in multiple mammalian species, including cattle [16, 20–23], sheep [18, 24, 25], goats [26], horses [27], mice [28], pigs [29], and humans [30]. These UF-EVs are implicated in several key reproductive processes within the uterus, such as the establishment of endometrial receptivity [21, 31], the elongation and implantation of the conceptus [16, 18, 24], and the regulation of maternal immune responses [22]. The protein [23, 32] and miRNA [21, 33] profiles of UF-EVs undergo dynamic changes throughout the bovine estrous cycle. Additionally, alterations in the miRNA profiles of UF-EVs in the presence of multiple embryos have been described, suggesting that the cargo of these vesicles is influenced by pre-hatching blastocysts [22].

Functionally, UF-EVs are internalized by bovine embryos and modulate their development and quality. Adding UF-EVs to the in vitro culture (IVC) medium significantly increases blastocyst yield [23] and enhances the developmental competence of somatic cell nuclear transfer embryos, as evidenced by improved blastocyst development and hatching [20]. Furthermore, we demonstrated that sequential supplementation of the IVC medium with oviduct fluid-EVs followed by UF-EVs enhances blastocyst quality. This improvement was evidenced by higher survival post-vitrification, increased total cell number, reduced lipid content, and modulation of lipid metabolism-related genes in blastocysts [34], potentially mediated by the miRNA cargo of EVs [33]. These findings emphasize the critical role of maternal UF-EVs in enhancing embryonic development and quality.

In summary, studies on embryo-derived EVs and UF-EVs suggest that the pre-hatching embryo and endometrium actively exchange EVs during pre-implantation. This exchange facilitates embryo-maternal cross-talk, supporting embryo development and endometrial receptivity. As we have recently shown, embryo presence can induce changes in the protein profile of bovine oviductal EVs on d 3.5 of pregnancy, as well as in in vitro-derived oviductal EVs [35]. However, how these signals modulate the protein content of bovine UF-EVs remains a key area of interest that requires further investigation. We hypothesized that the d 7 blastocyst induces specific alterations in the proteomic cargo of UF-EVs, which may influence endometrial receptivity and embryo development. Moreover, local effects of the embryo on the endometrium are challenging to study in vivo and highlight the need for an in vitro model to gain a deeper understanding of the complex maternal-embryonic cross-talk through EVs during the early stages of pregnancy.

Therefore, the present study aimed to: (i) characterize the protein content of UF-EVs from PREGNANT and CYCLIC heifers to identify embryo-induced changes in the UF-EV proteome, and to explore the biological processes potentially modulated by these embryo-maternal communication-driven alterations in UF-EV protein cargo in vivo; (ii) characterize the protein content of conditioned medium (CM)-EVs from endometrial explants cultured alone or co-cultured with d 7 blastocysts, or from d 7 blastocysts cultured alone to identify embryo-induced changes in the CM-EV proteome, and to explore the biological processes potentially modulated by these embryo-maternal communication-driven alterations in CM-EV protein cargo in vitro; and (iii) compare the EV protein profiles between in vivo and in vitro models (i.e., EXPL vs. CYCLIC and EXPL + EMB vs. PREGNANT) to identify shared and distinct features of embryo-maternal communication, and to evaluate the relevance of endometrial explants as a model system for studying EV-mediated signaling.

## Materials and methods

### Experimental design

**Fig. 1:**
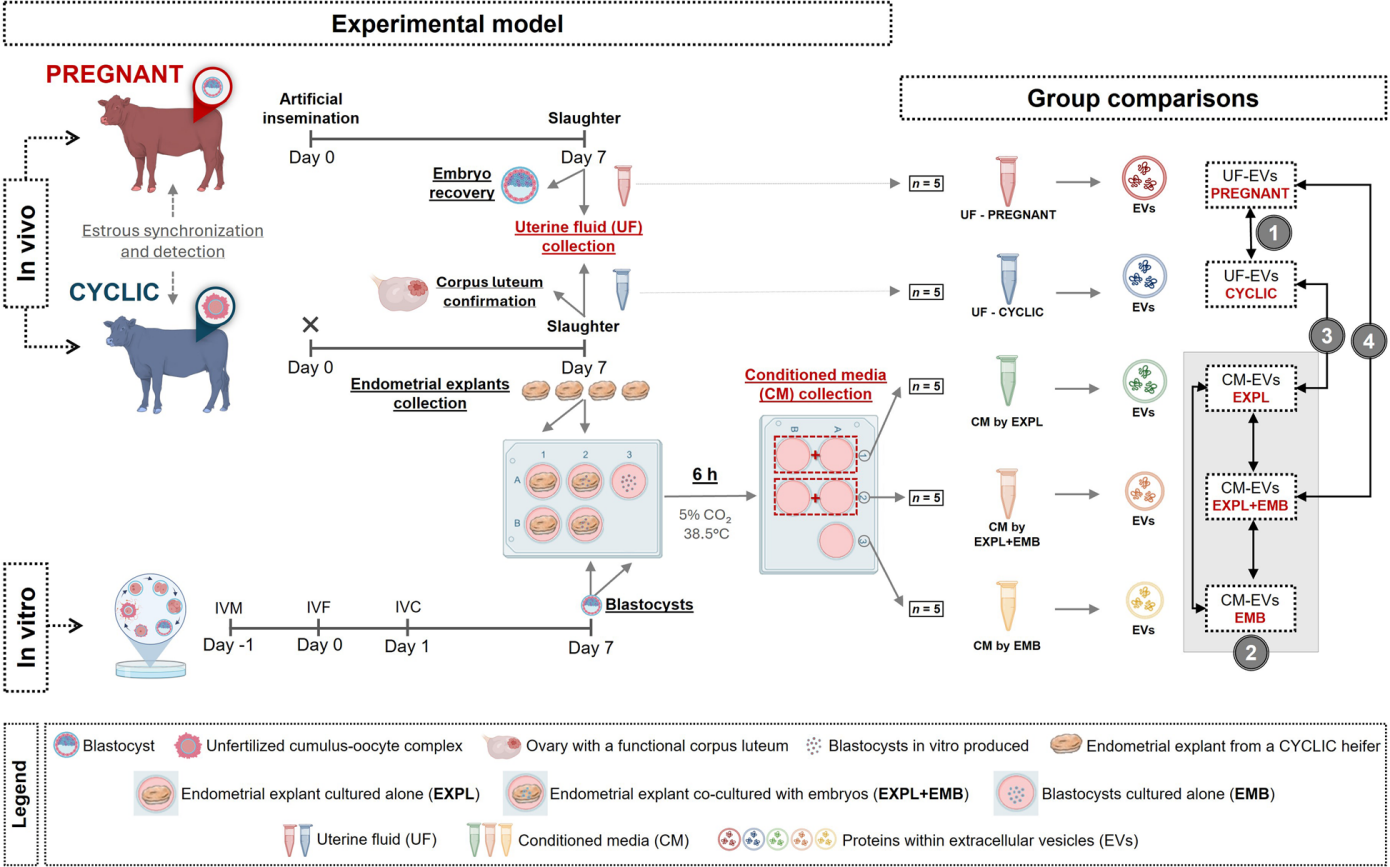
Experimental model and group comparisons. Heifers were synchronized and either artificially inseminated or not inseminated. At 7 days post-insemination, heifers were slaughtered and the uterine horn ipsilateral to the corpus luteum was flushed. Pregnancy was confirmed by recovering a blastocyst in inseminated heifers (PREGNANT group, *n* = 5), whereas the presence of a functional corpus luteum confirmed cyclic status in non‑inseminated heifers (CYCLIC group, *n* = 5). For the in vitro model, four 8 mm circular endometrial explants were collected from the ipsilateral horn of each CYCLIC heifer and cultured separately in 1 mL of protein-free synthetic oviduct fluid (SOF): two explants were cultured alone (EXPL group, *n* = 5), and two were co-cultured with five in vitro-produced bovine blastocysts (EXPL + EMB group, *n* = 5). Additionally, 50 in vitro-produced bovine blastocysts were cultured alone (EMB group, *n* = 5) in 500 μL of SOF. After 6 h, the conditioned medium (CM) was collected for EV isolation. For each cyclic heifer, CM from their two explants cultured alone was pooled to form one EXPL sample, and CM from their two explants co-cultured with blastocysts was pooled to form one EXPL + EMB sample. Each pool was treated as a single biological replicate per condition per cyclic heifer. The protein content of EVs was analyzed across the following four comparisons: (1) UF‑EVs from CYCLIC versus PREGNANT heifers; (2) CM‑EVs from EXPL, EXPL + EMB, and EMB; (3) UF‑EVs from CYCLIC heifers versus CM‑EVs from EXPL; and (4) UF‑EVs from PREGNANT heifers versus CM‑EVs from EXPL + EMB. Created in BioRender (https://BioRender.com/gizygdh)

The experimental design is shown in Fig. 1. We evaluated the protein content of EVs in four different comparisons:

1. UF-EVs from non-pregnant heifers (CYCLIC) compared to those from pregnant heifers (PREGNANT).
2. CM-EVs from endometrial explants cultured alone (EXPL) were compared to those from explants co-cultured with blastocysts (EXPL + EMB) and from blastocysts cultured alone (EMB).
3. UF-EVs from CYCLIC heifers were compared to EVs from the CM of endometrial explants cultured alone in vitro (EXPL).
4. UF-EVs from PREGNANT heifers were compared to EVs from the CM of endometrial explants co-cultured with blastocysts (EXPL + EMB) in vitro.

### In vivo model

#### Animals

All experimental procedures involving animals were approved by the Animal Research Ethics Committee of University College Dublin and licensed by the Health Products Regulatory Authority, Ireland, in accordance with Statutory Instrument No*.* 543 of 2012 under Directive 2010/63/EU on the Protection of Animals used for Scientific Purposes.

Crossbred beef heifers (*n* = 28 predominantly Charolais- and Limousin-cross); 804 ± 135 days old and 602.6 ± 54.3 kg (mean ± standard deviation) were synchronized using an 8-day intravaginal progesterone (P4) device (PRID, 1.55 g P4; Ceva Santé Animale). On the day of PRID insertion, each heifer received a 2-mL intramuscular injection of an analog of gonadotropin-releasing hormone (GnRH; Ovarelin, Ceva Santé Animale, equivalent to 100 μg gonadorelin). On the day before PRID removal, all heifers received a 5-mL intramuscular injection of an analog of prostaglandin F_2_ alpha (PGF_2_α; Enzaprost, Ceva Santé Animale, equivalent to 25 mg dinoprost) to induce luteolysis. Estrus was detected in heifers by visual observation performed by trained personnel every 4–6 h, starting 24 h after PRID removal. Heifers were randomly assigned to be either inseminated (*n* = 15) at detected estrus to generate pregnancies or not inseminated (*n* = 13) to generate cyclic controls. Heifers were inseminated twice, approximately 12 and 24 h after the onset of estrus. All heifers were slaughtered at a local abattoir approximately 7 d after artificial insemination.

#### UF collection from PREGNANT and CYCLIC heifers

Reproductive tracts were returned to the laboratory on ice within 3 h of slaughter. The presence of a functional corpus luteum CL was confirmed in all estrus-synchronized heifers by post-mortem ovarian inspection. The uterine horn ipsilateral to the CL was dissected and flushed with 10 mL of phosphate-buffered saline without Ca^2+^ and Mg^2+^ (PBS^−/−^). The presence of a blastocyst in the uterine flushing of inseminated heifers was used to confirm pregnancy (*n* = 12), while the uterine flushings from non-inseminated heifers were categorized as cyclic (*n* = 13). All flushings were centrifuged immediately for 7 min at 300 × *g* and 4 °C to remove cells. The obtained supernatants were then centrifuged for 30 min at 10,000 × *g* and 4 °C to remove cellular debris and conserved at −80 °C to be later processed for EV isolation. For downstream analyses, five heifers from the 12 confirmed pregnant and five from the 13 non-inseminated heifers were randomly selected to form the PREGNANT and CYCLIC groups, respectively.

### In vitro model

#### Preparation of endometrial explants

Four endometrial explants were obtained from each cyclic heifer, and prepared and cultured as described by Mathew et al. [36]. After flushing, the uterine horn ipsilateral to the CL was longitudinally opened on the anti-mesometrial side to expose the endometrium. Then, an 8-mm biopsy punch was used to dissect completely through the intercaruncular uterine tissue from the anterior portion of the uterine horn. Sterile scissors were then used to dissect the endometrium away from the myometrium. Once dissected, explants from the same animal were washed in Hanks’ Balanced Salt Solution (HBSS; Gibco, ThermoFisher Scientific) containing 1% antibiotic–antimycotic (ABAM; Gibco, 100×). Subsequently, these explants were individually cultured in a 24-well cell culture plate, with the endometrium side facing up, in wells containing 1 mL protein-free synthetic oviduct fluid (SOF) and under 5% CO_2_ at 38.5 °C in air with maximum humidity for 2 h before use.

#### Conditioned medium from endometrial explants and blastocysts

Before use, explants obtained as described above were transferred individually to new wells containing 1 mL equilibrated SOF. From the four endometrial explants obtained from each cyclic heifer, two were cultured in medium alone (EXPL), and two were co-cultured with five in vitro-produced bovine blastocysts each (EXPL + EMB) based on Passaro et al. [12], who showed that co-culture with at least five bovine blastocysts was necessary to induce transcriptomic changes in bovine endometrial explants. Also, five groups of 50 in vitro-produced blastocysts were cultured alone (EMB) in 500 μL of SOF each. A preliminary study showed that culturing 5 to 25 blastocysts for 6 h did not produce the minimum particle concentration required for reliable NTA detection (≥ 20 particles/frame). In contrast, 50 embryos consistently yielded sufficient EVs for NTA quantification and downstream analysis [37, 38]. Representative images of the EXPL, EXPL+EMB, and EMB groups are provided in Additional file 1.

All groups were cultured for 6 h at 5% CO_2_, 38.5 °C, and maximum humidity. A 6-h culture period was selected as it consistently induces embryo responsive alterations in endometrial explants and CM-derived EVs while preserving explant structural and functional integrity [12, 38–41]. After 6 h, the CM from all groups was collected. For each cyclic heifer, CM from their two explants cultured alone was pooled to form one EXPL sample, and CM from their two explants co-cultured with blastocysts was pooled to form one EXPL + EMB sample. A preliminary study demonstrated that pooling the CM from two explants per animal was necessary to achieve the minimum particle concentration required for reliable NTA detection (≥ 20 particles/frame) [37]. Each pool was treated as a single biological sample per condition for each cyclic heifer. CM from all groups were centrifuged for 7 min at 300 × *g* and 4 °C to remove cells. The obtained supernatant was then centrifuged for 30 min at 10,000 × *g* and 4 °C to remove cellular debris and the supernatant was stored at −80 °C to be later processed for EV isolation.

To ensure consistency between the in vitro and in vivo models, only explants from the same five CYCLIC heifers selected for the in vivo analyses were used. This resulted in five EXPL and five EXPL + EMB samples derived from these heifers. Additionally, CM from five independent EMB cultures was included in the downstream analyses of the in vitro model.

#### In vitro embryo production

Embryos were produced in vitro as previously described by Rizos et al. [1]. Briefly, bovine immature cumulus-oocyte complexes (COCs) were obtained by aspirating follicles from the ovaries of mature heifers slaughtered at a local abattoir. After selection, COCs were matured during 24 h in groups of 50/well in 500 µL maturation medium (TCM-199) supplemented with 10% of fetal calf serum (FCS) and 10 ng/mL epidermal growth factor (EGF) at 38.5 °C under an atmosphere of 5% CO_2_ in air with maximum humidity. Matured COCs were fertilized with frozen-thawed sperm from a bull of proven fertility at a concentration of 1× 10^6^ sperm/mL. Gametes were co-incubated in 500 µL of fertilization medium for 18–20 h at 38.5 °C, 5% CO_2_ in air with Maximum humidity. Presumptive zygotes were denuded by vortexing and cultured in 500 µL of SOF supplemented with 5% of EV-depleted FCS (dFCS) at 38.5 °C, under 5% CO_2_, 5% O_2_, and 90% N_2_ with maximum humidity. The dFCS was produced in our laboratory according to the protocol used by Leal et al. [34]. Briefly, heat-inactivated FCS (56 °C for 30 min) was ultra-centrifuged at 100,000 × *g* for 18 h at 4 °C using an Optima-L-100XP Beckman Coulter ultracentrifuge. The supernatant (dFCS) was collected, aliquoted, and stored at −20 °C for later use. Embryos of excellent or good quality [42] were recovered 7 d after fertilization at the blastocyst stage for subsequent use.

### EV isolation

Extracellular vesicles were isolated from the UF of five animals per group (*n* = 5 CYCLIC and *n* = 5 PREGNANT) and from five CM samples per group (*n* = 5 EXPL, *n* = 5 EXPL + EMB, and *n* = 5 EMB) following the protocol previously reported by our group [35].

This protocol is based on size-exclusion chromatography (SEC) using PURE-EV^®^ columns (HansaBioMed Life Sciences) [21], an effective method for separating EVs from circulating proteins without altering their structure or function [43], followed by ultrafiltration using Vivaspin^®^ Turbo 15 centrifugal concentrator (Sartorius, 100 K MWCO PES). Briefly, after discarding the buffer provided within the SEC column, the column was washed with 30 mL of PBS^**−/−**^**,** and then either UF (≈ 2 mL) or CM (≈ 2 mL) fluid samples were loaded onto the top of the SEC column. Once the sample was entirely within the column, 11 mL of PBS^**−/−**^was loaded, preventing the column from drying out. The EVs were collected in the 2.5 mL fraction after discarding the first 3 mL fraction. Subsequently, the 2.5 mL EV fraction was concentrated by ultrafiltration for 30 min at 2,000 × *g* and 4 °C, resulting in a final volume of 100 µL of concentrated EVs to be used later for EV characterization and proteomic analysis.

### EV characterization

Following the Minimal Information for Studies of Extracellular Vesicles 2018 guidelines [44], EVs from UF and CM were characterized using flow cytometry (FC), nanoparticle tracking analysis (NTA), and transmission electron microscopy (TEM) as previously described by Mazzarella et al. [35].

#### Flow cytometry

The analyses were conducted utilizing the high-sensitive flow cytometer CytoFLEX S (Beckman Coulter), equipped with violet (405 nm), blue (488 nm), yellow (561 nm), and red (638 nm) lasers. Recombinant EVs expressing green fluorescent protein (GFP, SAE0193, Merck) were used to verify the accuracy of the flow cytometer for EV detection and counting. The optical configuration was optimized to use side scatter (SSC) information from the 405-nm laser (vSCC). Both forward scatter (FSC) and SSC were set to logarithmic scale, with the fluorescence channels also adjusted to logarithmic scale. The analysis was restricted to events with FSC and SSC characteristics specific to EVs. Samples were analyzed using the low flow speed setting (10 μL/min) with a minimum acquisition of 10 × 10^3^ events per sample. Distilled water (filtered through a 0.1-μm filter) was used as the sheath fluid, and 0.1-μm-filtered phosphate-buffered saline (PBS) was employed to detect background noise. Two-minute washing steps with 0.1-μm-filtered distilled water were conducted between EV samples as described in Barranco et al. [45]. 10 μL EV sample was incubated with CellTrace CFSE (Thermofisher) for 30 min in darkness at 37 °C, a non-fluorescent probe that becomes fluorescent hydrolysis by active esterases present only in functional intact membrane structures, to discriminate intact EVs from membrane fragments. Tetraspanin antibodies anti-CD63-FITC and anti-CD81-APC (REA, Miltenyi Biotec) and anti-CD44-PerCP (Biolegend), with cross-reactivity with bovine species were used (30 min, RT in darkness), following the International Society of Extracellular Vesicles recommendations (MIFlowCyt-EV) [46].

#### Nanoparticle tracking analysis

The concentration and size distribution of EVs were analyzed using a NanoSight LM-10 system equipped with a CCD video camera and particle-tracking software NTA 3.1 Build 3.1.45 (NanoSight Ltd.). Five µL of UF-EVs or CM-EVs solution obtained after SEC were diluted in (1:10) with PBS^-/-^. PBS^-/-^ was used as a negative control. The NTA measurement conditions were detection thresholds 2 to 3, camera level 13, temperature 22 °C, and measurement time 60 s. Three recordings were performed for each sample.

#### Transmission electron microscopy

Transmission electron microscopy was exclusively employed to confirm the successful isolation of EVs following the established protocol. For that, 5 µL of UF-EVs or CM-EVs solution obtained after SEC were diluted (1:5) with PBS^-/-^ to perform the negative staining of EVs. A carbon-coated collodion 400 mesh nickel grid (Gilder) was floated for 2 min and stained with 2% uranyl acetate (Electron Microscopy Sciences) for 1 min for the negative staining. Grids were visualized in a JEOL JEM 1400 Flash electron microscope (operating at 100 kV). Micrographs were taken with a Gatan OneView digital camera at various magnifications.

### Proteomic analyses

Proteomic analysis was conducted as previously described by Mazzarella et al. [35].

#### In solution digestion

Protein was extracted in a sample containing 7 mol/L urea, 2 mol/L Thiourea, 4% CHAPS, and 5 mmol/L DTT, then digested following filter-aided sample preparation protocol described by Wisniewski et al. [47] with minor modifications. Trypsin, used to generate peptides through specific cleavage, was added at a trypsin: protein ratio of 1:20, and the mixture was incubated overnight at 37 °C, dried out in an RVC2 25 speedvac concentrator (Christ), and resuspended in 0.1% formic acid (FA). Peptides were desalted and resuspended in 0.1% FA using C18 stage tips (Millipore).

#### Mass spectrometry analysis

Samples were analyzed in a timsTOF Pro with PASEF (Bruker Daltonics) coupled online to an Evosep ONE liquid chromatograph (Evosep). A total of 200 ng were directly loaded onto the Evosep ONE and resolved using the 60 samples-per-day protocol. Protein identification and quantification were carried out using the label-free quantification (LFQ) method integrated into MaxQuant 1.6.17.0 software. Searches were carried out against a database consisting of *Bos* *taurus* entries from UniProt Swissprot + TrEMBL (downloaded on April 6, 2022), consisting of 117,111 entries. Carbamidomethylation of cysteines was set as a fixed modification, and oxidation of methionine and N-terminal acetylation of proteins were set as variable modifications. Two missed cleavages were allowed for trypsin digestion. Precursor and fragment tolerances of 20 ppm and 0.05 Da were considered for the searches, respectively.

A 1% false discovery rate (FDR) was applied at both the peptide-spectrum match (PSM) and protein levels. Only proteins identified with at least two peptides at FDR < 1% were considered for further analysis. Proteins were considered ‘identified’ when detected in at least three out of five samples in each experimental group and were considered ‘exclusive’ when detected in at least three out of five samples within one group and not detected in any sample within the other. The quantitative analysis of the in vitro model was performed in pairs: EXPL vs. EMB, EMB vs. EXPL + EMB, and EXPL vs. EXPL + EMB. Initially, differentially abundant proteins (DAPs) were identified for each comparison. Then, to isolate proteins specifically associated with embryo-maternal interactions, those proteins also differentially abundant in the EXPL vs. EMB comparison, where no embryo-maternal interaction occurs, were excluded. Subsequently, to exclude proteins whose differential abundance arises solely from the individual effects of the explant (EXPL) or the embryo (EMB), only proteins differentially abundant in both the EXPL vs. EXPL + EMB and EMB vs. EXPL + EMB comparisons were considered. The differential abundance of these common proteins was attributed to the synergistic effects of the co-culture of explants with embryos (EXPL + EMB), reflecting embryo-maternal communication, and was subsequently used for downstream analysis. The strategy used to identify differentially abundant proteins associated with embryo-maternal interactions in vitro is illustrated in Additional file 2.

### Statistical and bioinformatics analysis

#### EV characterization

In vivo and in vitro data were tested for outliers using the ROUT test and for normality using the Shapiro–Wilk test. Normality was confirmed, and in vivo*,* data were analyzed using Student’s *t*-test, while in vitro data were analyzed using one-way ANOVA followed by Tukey’s test. Statistical analyses were performed using GraphPad Prism 10. For all analyses, *P* ≤ 0.05 was considered significant.

#### Proteomics

Protein abundance data were analyzed using Student’s *t*-test. For all analyses, *P* ≤ 0.05 was considered significant for further analyses and discussion. Peak area data were transformed using log2 for graphical representation. Principal component analysis (PCA) was generated by Metaboanalyst 6.0 (https://www.metaboanalyst.ca). Venn diagrams were constructed using Venny 2.1 (https://bioinfogp.cnb.csic.es/tools/venny/). Molecular function, biological processes, cellular processes, protein class, and identification of biological pathways of the proteins were evaluated using the PANTHER 18.0 Classification System (https://PANTHERdb.org/) with *Bos taurus* as the selected organism [48]. Metascape Membership tool v3.5.20240101 (https://metascape.org) was used to identify significant enrichment (*P* ≤ 0.05) matching the term “embryo development” [49].

## Results

### EV characterization

EVs isolated from both in vivo and in vitro models were characterized regarding their size and concentration using NTA, their EV marker expression (CD63, CD81, and CD44) through FC, and their morphology by TEM.

#### In vivo model

**Fig. 2:**
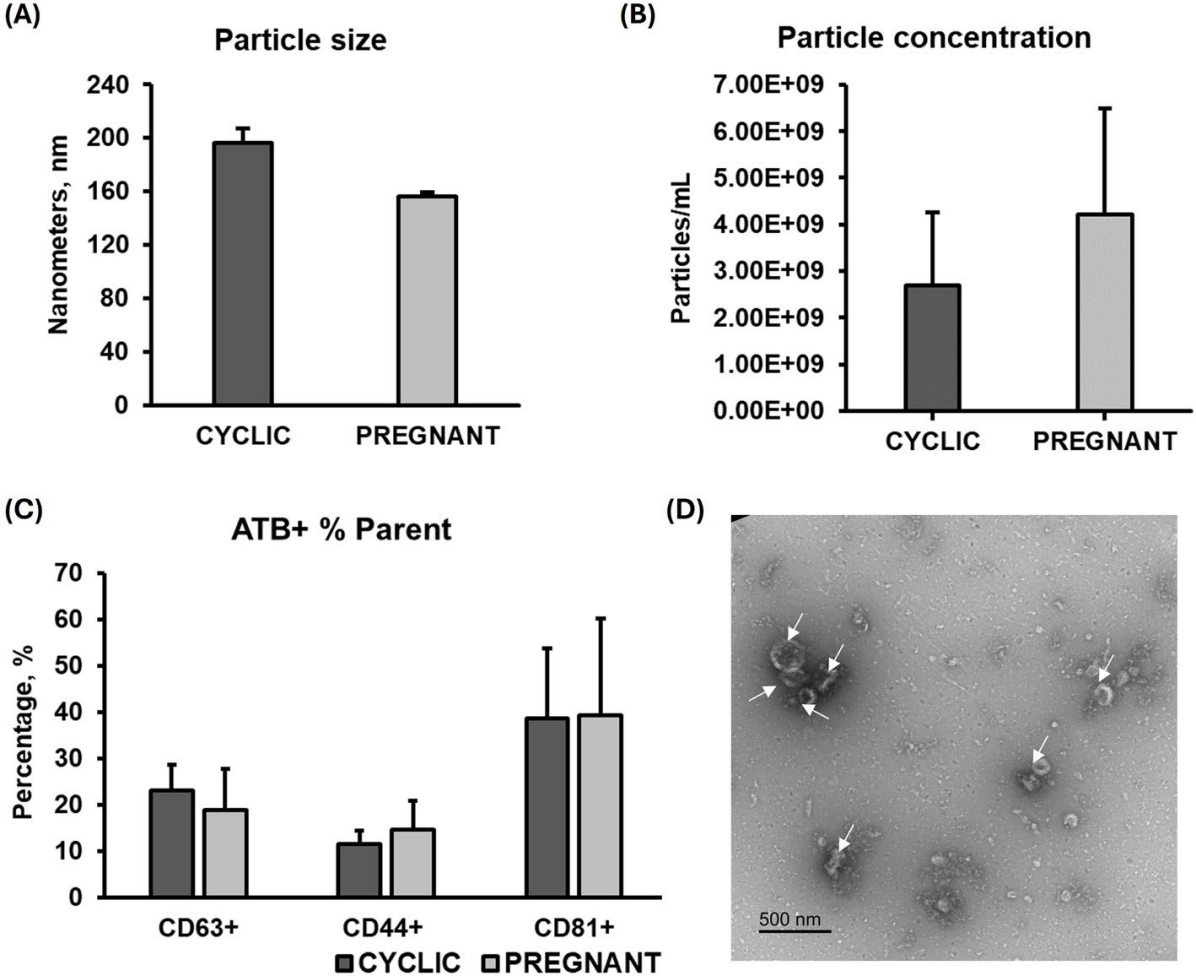
Characterization of uterine fluid extracellular vesicles (UF-EVs). Nanoparticle tracking analysis indicated no difference in particle size (**A**) or concentration (**B**) between the groups CYCLIC and PREGNANT. **C** Flow cytometry detected the presence of CD63, CD81, and CD44 markers in both groups. **D** Transmission electron microscopy images of UF-EVs, representative of both groups, revealed cup-shaped particles with sizes characteristic of EVs*.* Error bars represent the standard error of the mean. White arrows indicate EVs

The NTA analysis demonstrated no significant difference in particle size between the CYCLIC (196 ± 11.25 nm) and PREGNANT (156.4 ± 2.7 nm) groups (Fig. 2A), nor in particle concentration (CYCLIC: 2.70 × 10^9^ ± 1.55 × 10^9^ particles/mL and PREGNANT: 4.21 × 10^9^ ± 2.29 × 10^9^ particles/mL; Fig. 2B). The NTA negative control showed zero particles per frame (Additional file 3A). The presence of EVs was corroborated by FC, which identified CD63, CD81, and CD44 positive events in both groups (Fig. 2C). No differences in the percentage of CD63⁺ (CYCLIC: 23% ± 5.66% and PREGNANT: 19% ± 8.91%), CD44⁺ (CYCLIC: 12% ± 2.90% and PREGNANT: 15% ± 6.13%), and CD81⁺ (CYCLIC: 39% ± 15.11% and PREGNANT: 39% ± 20.83%) EVs among the groups were observed. TEM images displayed cup-shaped particles with sizes characteristic of EVs in the UF (Fig. 2D), while no particles were observed in the negative control (Additional file 3B). Thus, we confirmed the presence of EVs in the UF of both CYCLIC and PREGNANT heifers and validated the effectiveness of the isolation protocol.

#### In vitro model

**Fig. 3:**
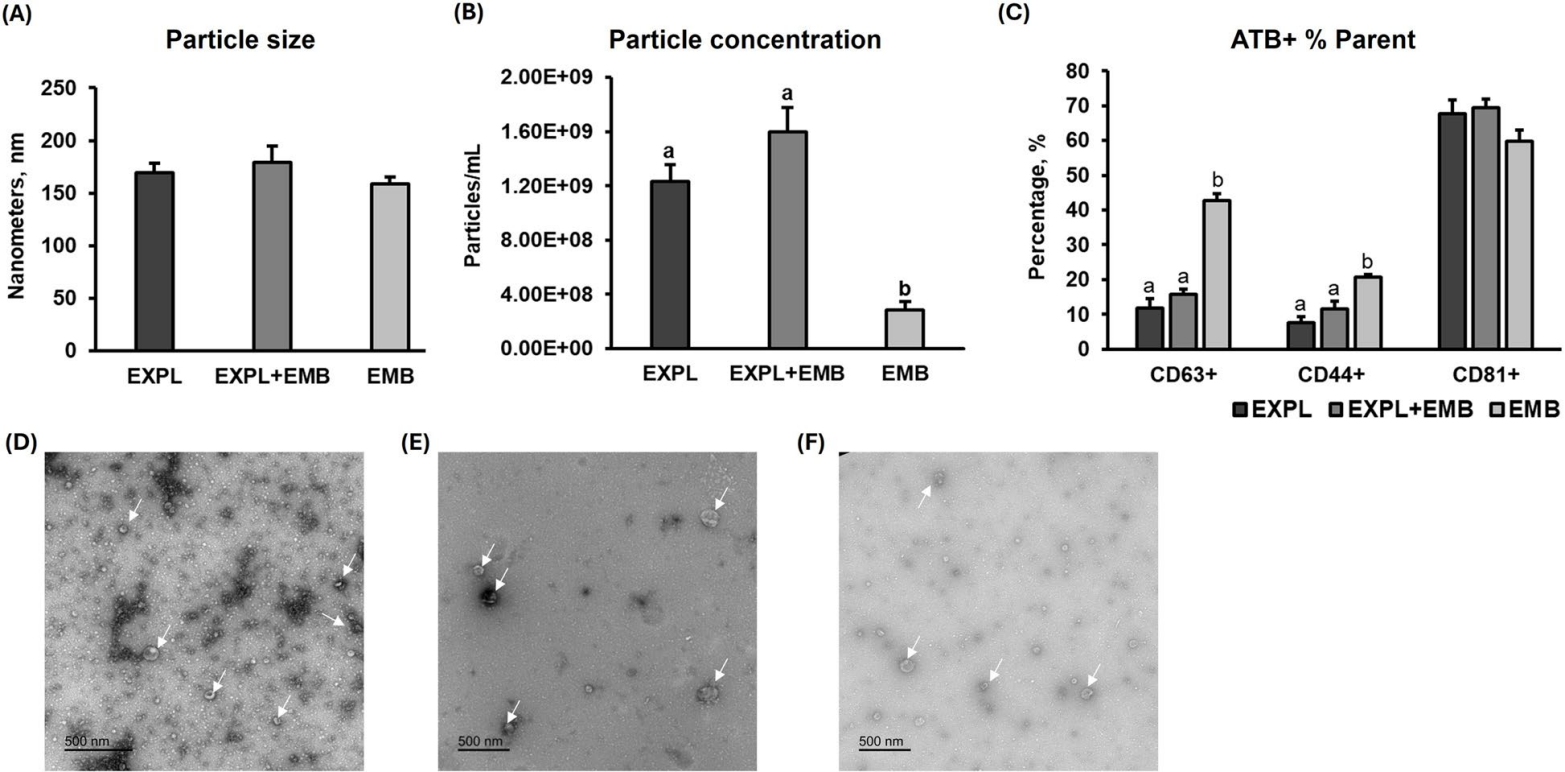
Characterization of conditioned medium (CM) extracellular vesicles (EVs). Nanoparticle tracking analysis revealed no significant differences in particle size (**A**) among the explants cultured alone (EXPL), explants co-cultured with embryos (EXPL + EMB), and embryos cultured alone (EMB) groups, while particle concentration was lower in the CM from EMB in comparison to EXPL and EXPL + EMB (**B**). Flow cytometry confirmed the presence of CD63, CD81, and CD44 markers in all groups (**C**). Transmission electron microscopy images showed cup-shaped particles with sizes characteristic of EVs in the CM from EXPL (**D**), EXPL + EMB (**E**), and EMB (**F**). Error bars represent the standard error of the mean. White arrows indicate EVs. Different letters indicate significant differences (*P* ≤ 0.05)

The NTA analysis indicated no significant differences in particle size among the EXPL, EXPL + EMB, and EMB groups (EXPL: 169 ± 8.78 nm, EXPL + EMB: 179 ± 16.02 nm, EMB: 159 ± 6.10 nm; Fig. 3A). Particle concentration was lower in the CM from EMB (EXPL: 1.23 × 10^9^ ± 1.27 × 10^8^ particles/mL, EXPL + EMB: 1.60 × 10^9^ ± 1.82 × 10^8^ particles/mL and EMB: 2.84 × 10^8^ ± 6.28 × 10^7^ particles/mL; Fig. 3B). The NTA negative control showed zero particles per frame (Additional file 3A). EV presence was confirmed via FC, identifying the CD63, CD81, and CD44 markers across all three groups (Fig. 3C). A significant difference in the percentage of CD63⁺ and CD44⁺ EVs among the groups was observed (*P* = 0.0001 and *P* = 0.0038, respectively). The EMB group exhibited a higher percentage of CD63⁺ EVs (43% ± 2.11%) compared to EXPL (12% ± 2.63%) and EXPL + EMB (16% ± 1.41%). Similarly, EMB group showed a higher percentage of CD44⁺ EVs (21% ± 0.90%) than EXPL (8% ± 1.68%) and EXPL + EMB (12% ± 2.13%). Regarding CD81⁺ EVs, no differences were found between the groups (EXPL: 68% ± 3.87%, EXPL + EMB: 70% ± 2.45% and EMB: 60% ± 3.11%). TEM images showed cup-shaped particles with sizes characteristic of EVs in the CM from EXPL, EXPL + EMB, and EMB groups (Fig. 3D–F), while no particles were observed in the negative control (Additional file 3B). Therefore, we confirmed the presence of EVs in the CM and validated the effectiveness of the isolation protocol.

### Qualitative and quantitative characterization of proteins

#### In vivo model

**Fig. 4:**
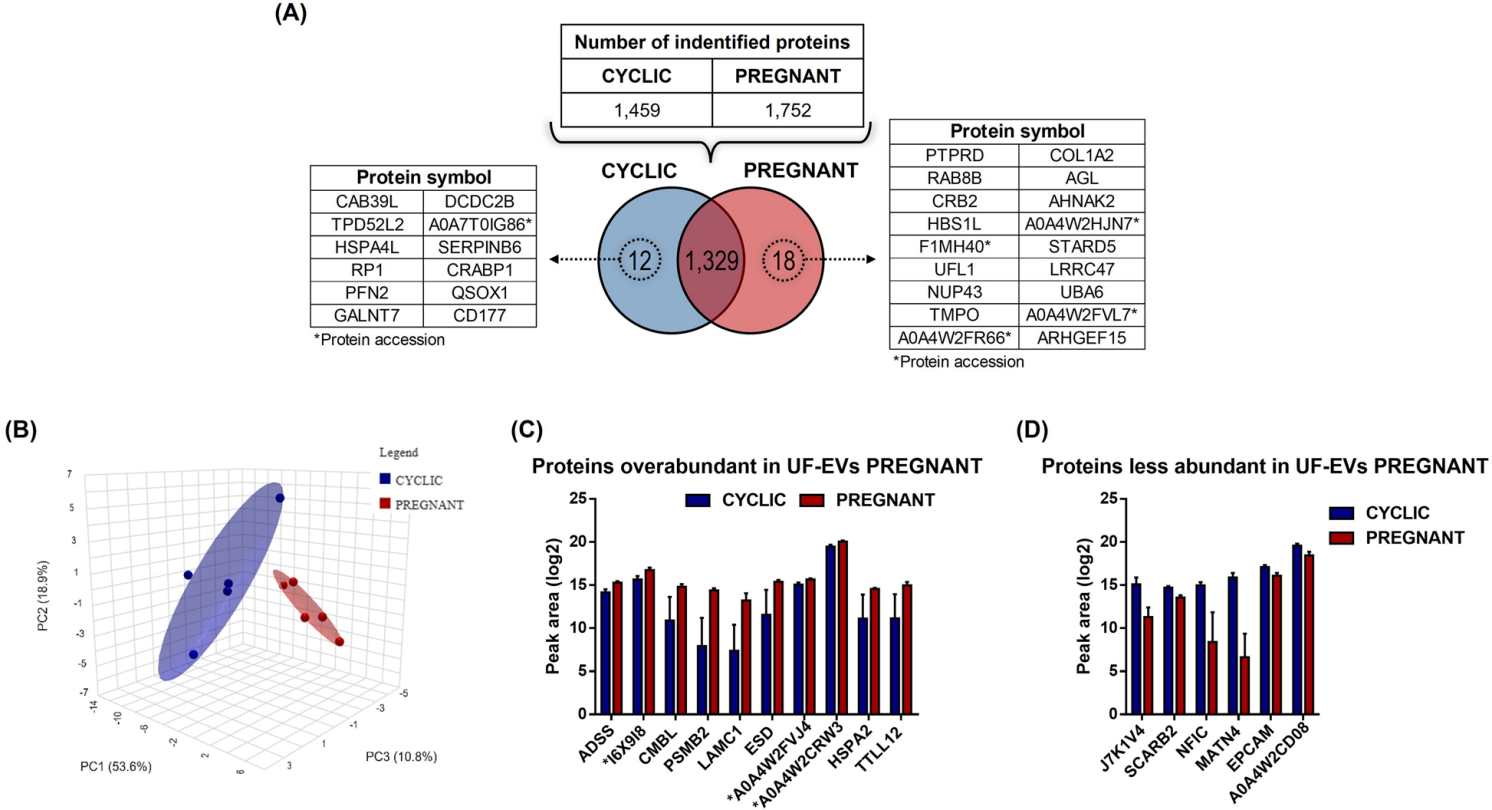
Protein profile of uterine fluid extracellular vesicles (UF-EVs) from CYCLIC and PREGNANT heifers. **A** The table indicates the number of proteins identified in each group, and the Venn diagram represents the 1,329 proteins common to both, the 12 proteins exclusively detected in CYCLIC and 18 proteins exclusively detected in PREGNANT heifers. **B** Principal Component Analysis of differentially abundant proteins. **C** Ten proteins were overabundant in UF-EVs from PREGNANT compared to CYCLIC heifers. **D** Six proteins were less abundant in UF-EVs from PREGNANT compared to CYCLIC heifers. Proteins were considered ‘identified’ if detected in at least three out of five replicates and considered ‘exclusive’ if detected in at least three out of five replicates within one group but absent in all samples of other groups. Error bars represent the standard error of the mean. *P *≤ 0.05 was considered as significant

We identified 1,459 proteins in the UF-EVs of CYCLIC and 1,752 proteins in the UF-EVs from PREGNANT heifers (Additional files 4A and 4B, respectively). Of these, 1,329 proteins were commonly identified between the two groups, whereas 12 were exclusive to UF-EVs from CYCLIC and 18 were exclusive to UF-EVs from PREGNANT heifers (Fig. 4A). Additionally, the PCA plot (Fig. 4B) from the DAPs revealed two distinct clusters of CYCLIC and PREGNANT heifers. Among the 1,329 identified in both groups, 16 proteins were differentially abundant (*P* ≤ 0.05; Additional file 4C): six proteins were less abundant, and 10 were more abundant in PREGNANT vs. CYCLIC heifers (Fig. 4C and D).

Among the top 30 most abundant proteins in CYCLIC heifers (Table 1A), 19 were ribosomal proteins (RPS8, RPL18, RPL7A, RPL7, RPL6, RPL19, RPS6, RPL36, RPS9, E1BK63, RPLP0, RPS2, RPL31, RPL24, RPL13A, RPS16, RPL10A, RPS25 and RPL27). Similarly, in PREGNANT heifers (Table 1B), 18 of the 30 most abundant proteins were ribosomal proteins (PIK3C2A, RPS2, RPS24, RPS4, RPS6, RPS8, RPS9, RPLP0, Q862L6, RPL13A, RPL31, RPL36, RPL6, RPL7A, RPL10A, RPL18, RPL19, and RPL7). The top 100 most abundant proteins in UF-EVs from CYCLIC and PREGNANT heifers are available in Additional file 4D. 

**Table 1:** Top 30 most abundant proteins identified in vivo

**Protein accession**	**Symbol**	**Description**	**Peak area (log2)**
(**A**) Top 30 most abundant proteins in UF-EVs from CYCLIC heifers			
A0A4W2FD79	ZNF638	Zinc finger protein 638	23.70
E1BAU6	INPP5E	Inositol polyphosphate-5-phosphatase E	22.73
A0A4W2EXX8	RPS8	40S ribosomal protein S8	22.22
A0A4W2EFE8	RPL18	Ribosomal protein L18	21.88
A0A4W2GZL4	IL1RAP	Interleukin 1 receptor accessory protein	21.66
Q2TBQ5	RPL7A	60S ribosomal protein L7a	21.60
A0A4W2GAA4	RPL7	Ribosomal protein L7	21.60
A0A4W2DYQ2	ACTB	Actin beta	21.51
A0A4W2GH10	ANPEP	Aminopeptidase	21.50
A0A4W2HYA4	EZR	Ezrin	21.42
A0A4W2E7H4	RPL6	60S ribosomal protein L6	21.39
A0A4W2E0U9	RPL19	Ribosomal protein L19	21.23
A0A4W2EMD8	RPS6	40S ribosomal protein S6	21.06
A0A4W2D0E5	RPL36	60S ribosomal protein L36	21.02
A0A4W2D2Y6	RPS9	40S ribosomal protein S9	21.02
E1BK63	─	Ribosomal protein L15	21.02
A0A4W2DVZ1	TUBB	Tubulin beta chain	20.99
A0A4W2H475	RPLP0	60S acidic ribosomal protein P0	20.98
Q28042	OVGP1	Oviduct-specific glycoprotein (Fragment)	20.90
F1MQ37	MYH9	Myosin heavy chain 9	20.89
A0A452DIA7	RPS2	40S ribosomal protein S2	20.87
A0A4W2CVQ1	ANXA2	Annexin	20.82
A0A4W2FQP8	RPL31	60S ribosomal protein L31	20.79
A0A4W2F392	RPL24	Ribosomal protein L24	20.79
A0A4W2CJG3	RPL13A	60S ribosomal protein L13a	20.76
A0A4W2HV66	RPS16	Ribosomal protein S16	20.76
P09487	ALPL	Alkaline phosphatase, tissue-nonspecific isozyme	20.74
A0A4W2HK63	RPL10A	Ribosomal protein	20.69
A0A4W2EYV4	RPS25	40S ribosomal protein S25	20.67
A0A4W2CDD8	RPL27	60S ribosomal protein L27	20.67
(**B**) Top 30 most abundant proteins in UF-EVs from PREGNANT heifers			
A0A4W2FD79	ZNF638	Zinc finger protein 638	23.45
E1BAU6	INPP5E	Inositol polyphosphate-5-phosphatase E	22.51
A0A4W2EXX8	RPS8	40S ribosomal protein S8	22.19
A0A4W2GH10	ANPEP	Aminopeptidase	22.10
A0A4W2DYQ2	ACTB	Actin beta	21.93
A0A4W2EFE8	RPL18	Ribosomal protein L18	21.72
A0A4W2GAA4	RPL7	Ribosomal protein L7	21.67
A0A4W2C549	LGALS3BP	Galectin-3-binding protein	21.46
A0A4W2DVZ1	TUBB	Tubulin beta chain	21.34
F1MZ85	VCAN	Versican core protein	21.28
Q2TBQ5	RPL7A	60S ribosomal protein L7a	21.22
A0A4W2E7H4	RPL6	60S ribosomal protein L6	21.19
A0A4W2CVQ1	ANXA2	Annexin	21.15
F1MQ37	MYH9	Myosin heavy chain 9	21.13
A0A4W2E0U9	RPL19	Ribosomal protein L19	21.10
Q862L6	─	60S ribosomal protein L12 (Fragment)	21.05
A0A4W2HYA4	EZR	Ezrin	21.03
A0A452DIA7	RPS2	40S ribosomal protein S2	21.03
A0A4W2GZL4	IL1RAP	Interleukin 1 receptor accessory protein	21.00
A0A4W2H475	RPLP0	60S acidic ribosomal protein P0	21.00
A0A4W2D2Y6	RPS9	40S ribosomal protein S9	20.97
A0A4W2EMD8	RPS6	40S ribosomal protein S6	20.80
A0A4W2CJG3	RPL13A	60S ribosomal protein L13a	20.80
P09487	ALPL	Alkaline phosphatase, tissue-nonspecific isozyme	20.79
A0A4W2F5Z1	RPS24	40S ribosomal protein S24	20.74
A0A4W2HK63	RPL10A	Ribosomal protein	20.73
P79103	RPS4	40S ribosomal protein S4	20.68
A0A4W2FQP8	RPL31	60S ribosomal protein L31	20.68
A0A4W2C999	PIK3C2A	40S ribosomal protein S13	20.64
A0A4W2D0E5	RPL36	60S ribosomal protein L36	20.49

**Fig. 5:**
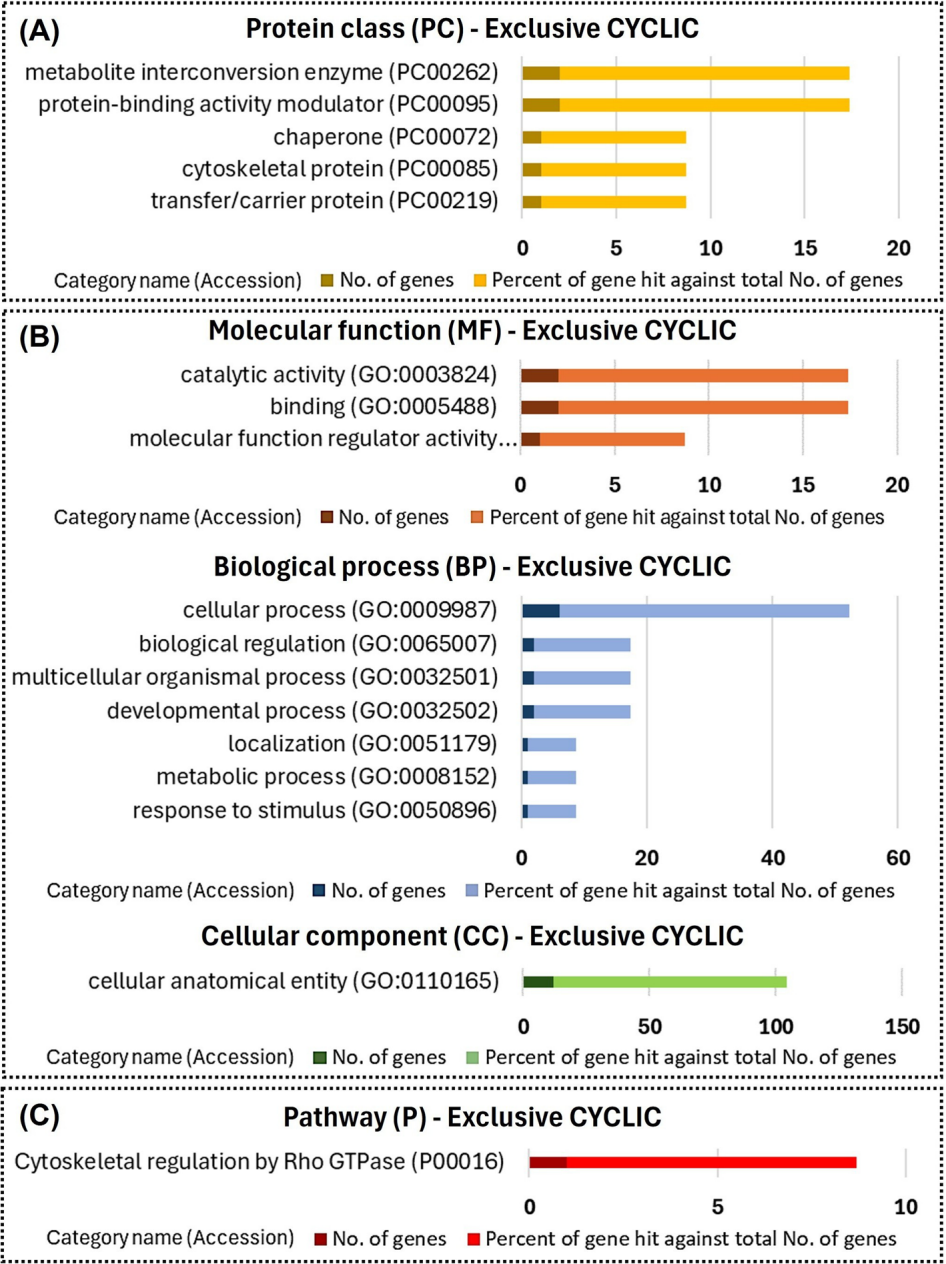
Functional enrichment of the proteins exclusive to uterine fluid extracellular vesicles of CYCLIC heifers. Protein class (**A**), Gene Ontology (**B**), and pathways (**C**) identified using the PANTHER 18.0 Classification System (https://pantherdb.org/). Darker bars indicate the number of genes associated with each category name, while lighter bars represent the percentage of these genes relative to the total number of genes in that category

**Fig. 6:**
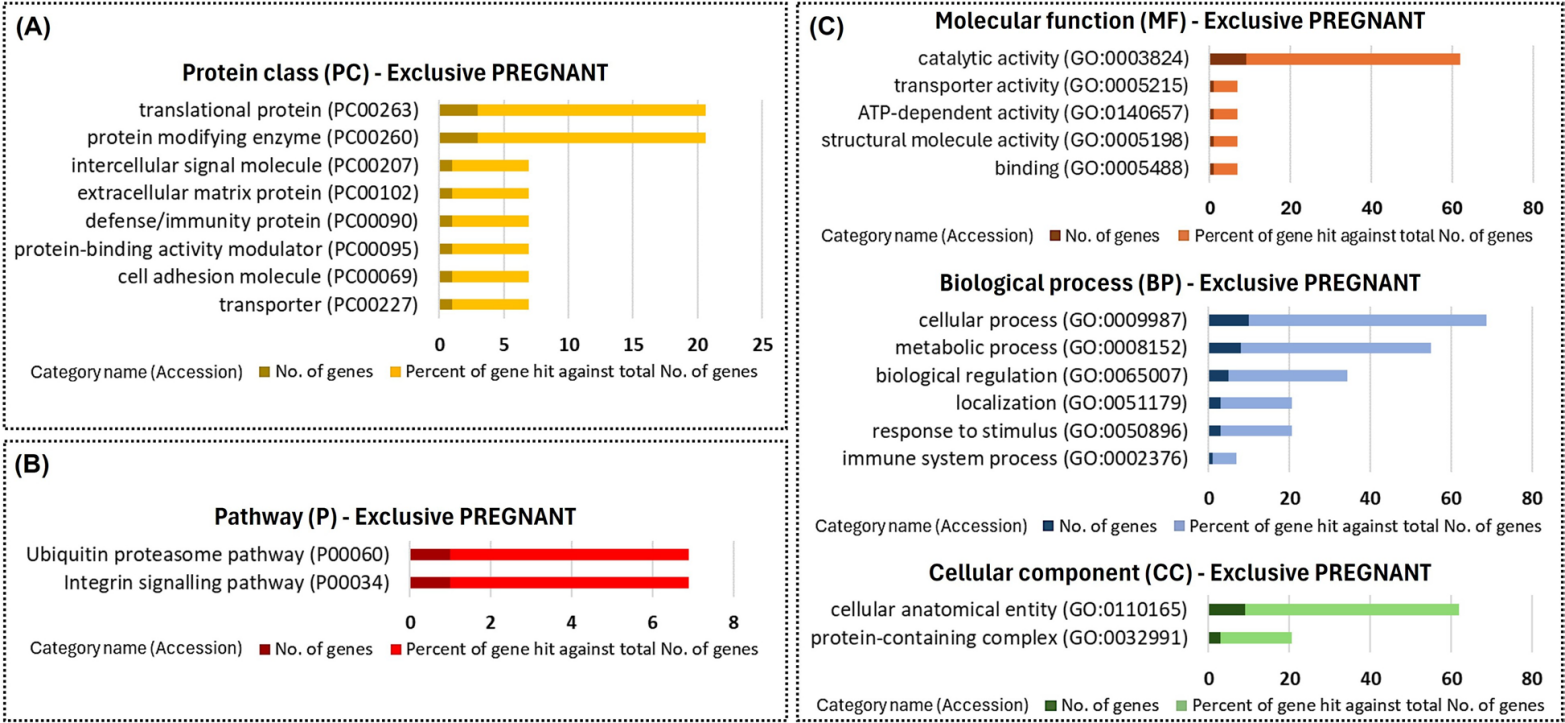
Functional enrichment of the proteins exclusive to uterine fluid extracellular vesicles of PREGNANT heifers. Protein class (**A**), Gene Ontology (**B**), and pathways (**C**) identified using the PANTHER 18.0 Classification System (https://pantherdb.org/). Darker bars indicate the number of genes associated with each category name, while lighter bars represent the percentage of these genes relative to the total number of genes in that category

Functional enrichment using the PANTHER database indicated that among proteins exclusive to UF-EVs of CYCLIC heifers were proteins with enzymes related catalytic activity (Fig. 5A and B), and proteins involved in the cytoskeletal regulation pathway (Fig. 5C). Among the proteins exclusive to UF-EVs in PREGNANT heifers were translational proteins and protein-modifying enzymes (Fig. 6A). These proteins were also involved in biological processes including cellular and metabolic activities (Fig. 6C) and were components of pathways such as the integrin signaling pathway (Fig. 6B).

**Fig. 7:**
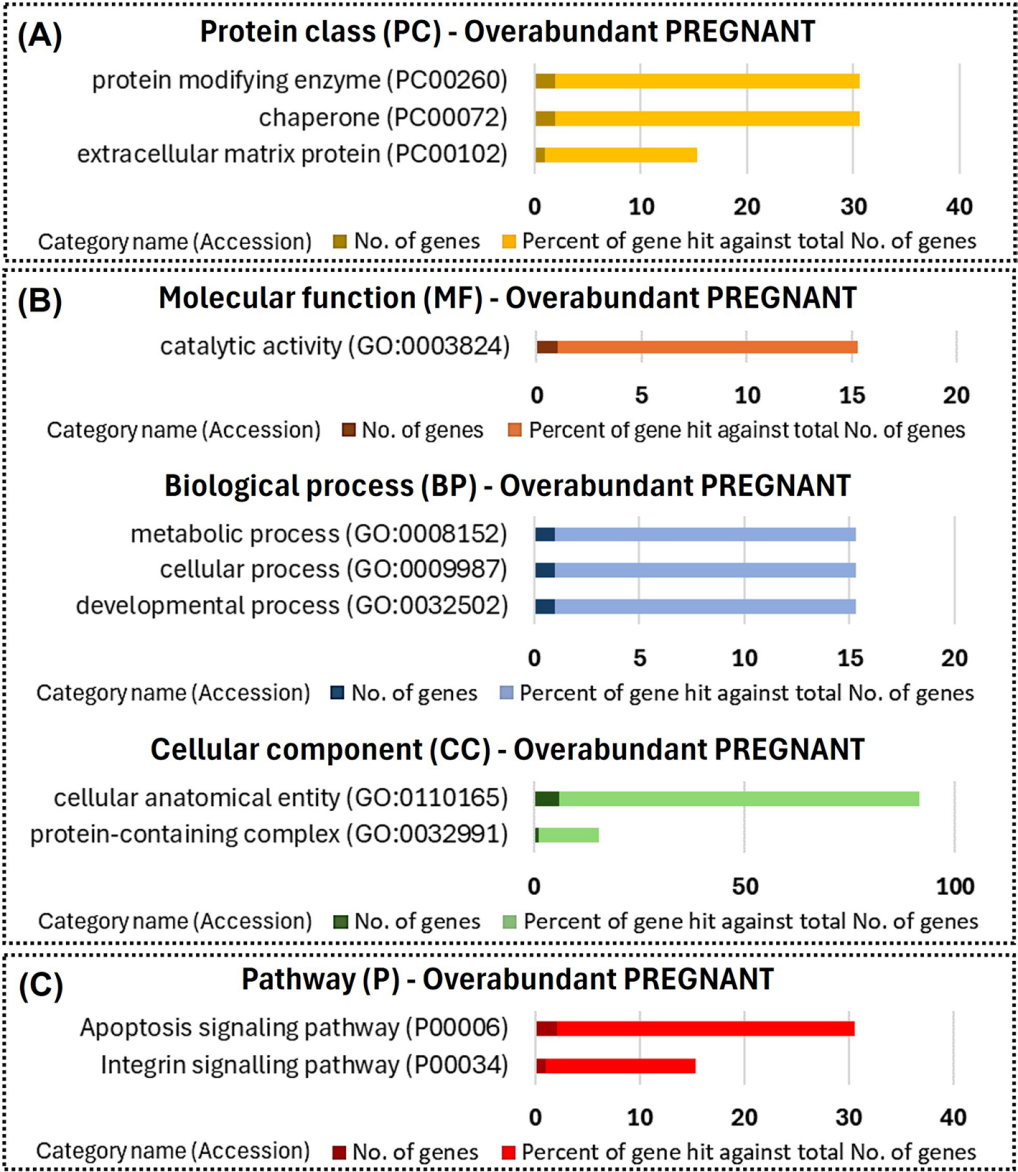
Functional enrichment of proteins overabundant in uterine fluid extracellular vesicles from PREGNANT versus CYCLIC heifers. Protein class (**A**), Gene Ontology (**B**) and pathways (**C**) identified using the PANTHER 18.0 Classification System (https://pantherdb.org/). Darker bars indicate the number of genes associated with each category name, while lighter bars represent the percentage of these genes relative to the total number of genes in that category

**Fig. 8:**
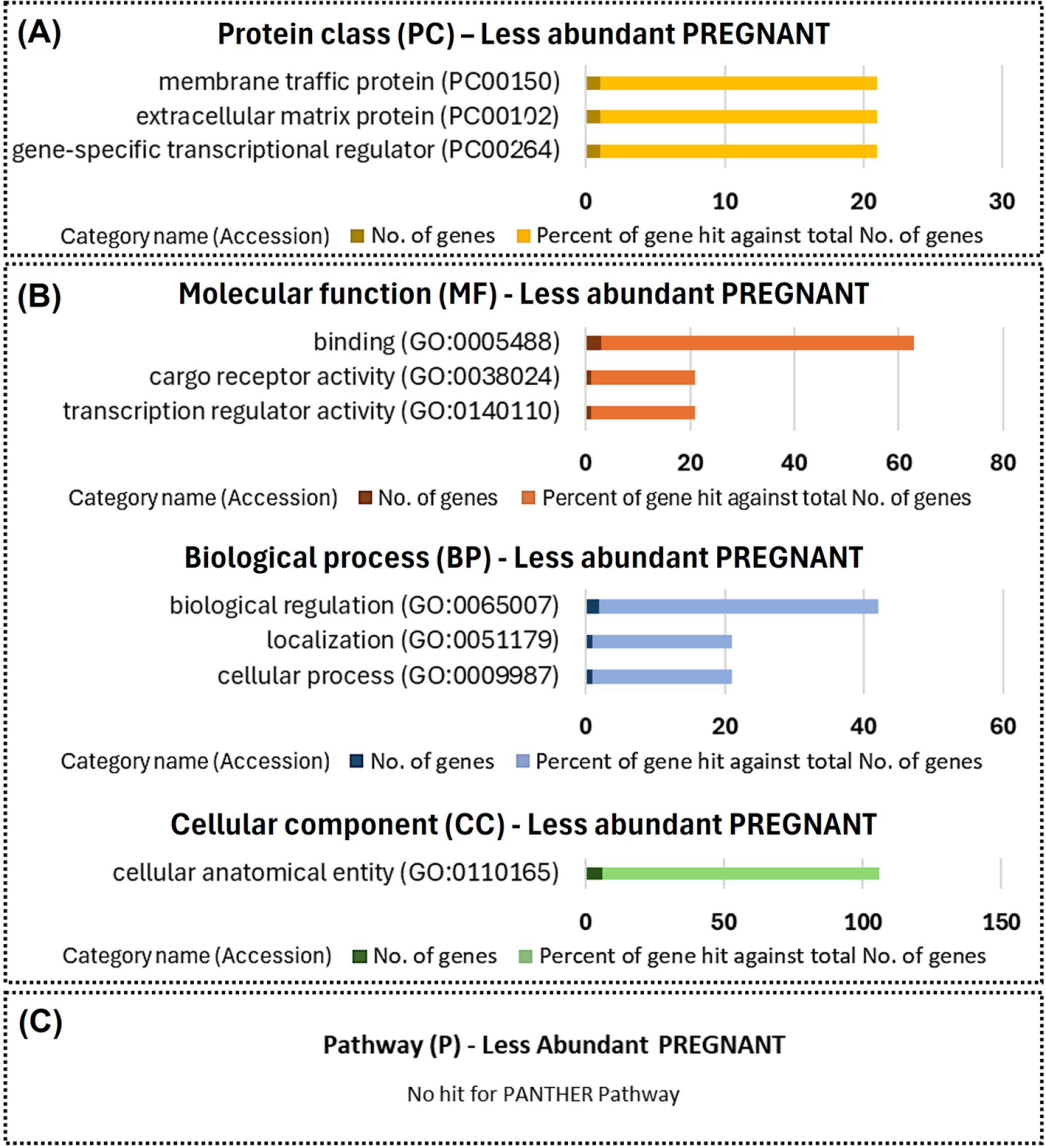
Functional enrichment of proteins less abundant in uterine fluid extracellular vesicles from PREGNANT versus CYCLIC heifers. Protein class (**A**), gene ontology (**B**), and pathways (**C**) identified using the PANTHER 18.0 Classification System (https://pantherdb.org/). Darker bars indicate the number of genes associated with each category name, while lighter bars represent the percentage of these genes relative to the total number of genes in that category

Overabundant proteins in PREGNANT heifers compared to CYCLIC heifers were related to protein-modifying enzymes (Fig. 7A). The less abundant proteins were associated with membrane trafficking, the extracellular matrix, and transcriptional regulation (Fig. 8A). Overabundant proteins were mainly associated with metabolic, cellular, and developmental processes, and were involved in pathways such as apoptosis and integrin signaling (Fig. 7B and C). Additionally, the less abundant proteins were related to biological regulation and cellular processes (Fig. 8B). No pathways were identified as modulated by the less abundant proteins in PREGNANT heifers (Fig. 8C).

#### In vitro model

**Fig. 9:**
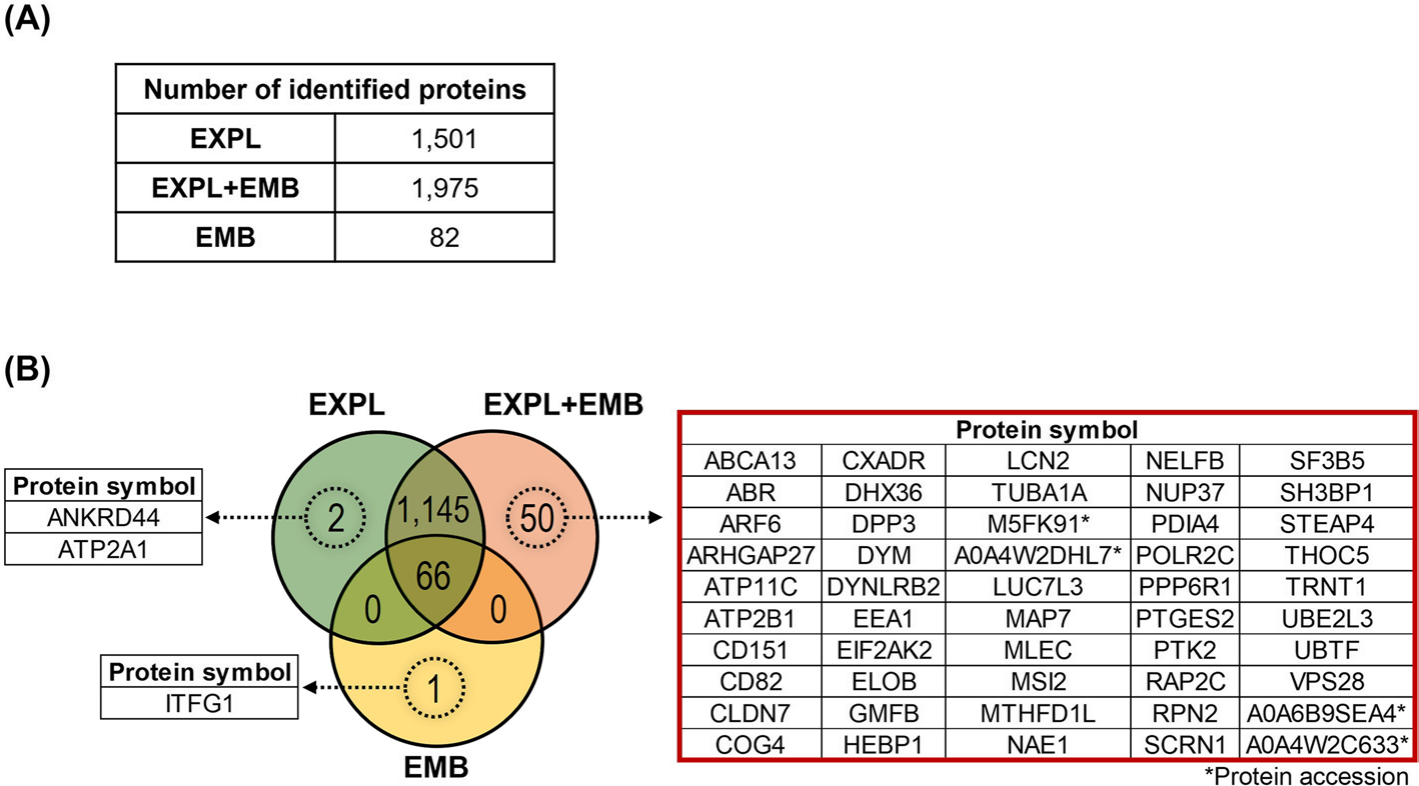
Protein profile of in vitro-derived extracellular vesicles (EVs). EVs were isolated from conditioned medium (CM) following the culture of endometrial explants in the absence (EXPL) or presence (EXPL + EMB) of blastocysts, or from blastocysts cultured alone (EMB). **A** Table representing the 1,501 proteins identified in the CM-EVs from ExpL, 1,975 proteins identified in ExpL + Emb, and 82 proteins identified in EMB. **B** Venn diagram represents the number of proteins associated with CM-EVs from EXPL, EXPL + EMB, and EMB. The red box indicates the list of the 50 proteins identified as only present when there is an interaction between the endometrium and the embryo in vitro. Proteins were considered ‘identified’ if detected in at least three out of five replicates and considered ‘exclusive’ if detected in at least three out of five replicates within one group but absent in all samples of other groups

We identified 1,501 proteins in the CM-EVs from EXPL, 1,975 from EXPL + EMB, and 82 from EMB (Fig. 9A, Additional files 5A, 5B and 5C, respectively). Of these, 66 proteins were commonly identified among the three groups, 1,145 were common to EXPL and EXPL + EMB but not to EMB (Fig. 9B). Two proteins were unique to EXPL (ANKRD44 and ATP2A1), and one was unique to EMB (ITFG1). Additionally, 50 proteins were identified as being exclusively present in the EXPL + EMB group (Fig. 9B, red box) when there is an interaction between the endometrium and the embryo in vitro.

Among the top 30 most abundant proteins in EXPL (Table 2A) and EXPL + EMB (Table 2B) were proteins such as zinc finger protein 638 (ZNF638), histones (H4 and H2B), inositol polyphosphate-5-phosphatase E (INPP5E), actins (ACTB, and ACTC1), tubulins (TUBB, TUBA1D and TUBB4B) and heat shock protein (HSP90AA1 and HSPA8). In EMB, the most abundant proteins include inositol polyphosphate-5-phosphatase E (INPP5E), zinc finger proteins (ZNF638), and keratins (KRT76, KRT18, KRT75, and KRT8) (Table 2C). The top 100 most abundant proteins in CM-EVs from EXPL, EXPL + EMB, and EMB are available in Additional file 5D.

**Table 2:** Top 30 most abundant proteins in vitro

**Protein accession**	**Symbol**	**Description**	**Peak area (log2)**
(**A**) Top 30 most abundant proteins in CM-EVs from EXPL			
A0A4W2FD79	ZNF638	Zinc finger protein 638	23.77
A0A4W2HPP0	─	Uncharacterized protein	23.23
P62803	─	Histone H4	23.23
A0A4W2HHA6	─	Histone H2B	23.10
E1BAU6	INPP5E	Inositol polyphosphate-5-phosphatase E	23.03
A0A4W2DYQ2	ACTB	Actin beta	22.94
A0A3Q1MDT7	─	Histone H4	22.68
Q3ZC07	ACTC1	Actin, alpha cardiac muscle 1	22.06
A0A4W2DVZ1	TUBB	Tubulin beta chain	21.51
A0A4W2H221	FGA	Fibrinogen alpha chain	21.25
P10096	GAPDH	Glyceraldehyde-3-phosphate dehydrogenase	21.24
F1MGU7	FGG	Fibrinogen gamma-B chain	21.23
F6S1Q0	KRT18	Keratin 18	21.18
A0A452DIF5	H1-2	H1.2 linker histone, cluster member	21.15
A0A4W2F0F2	TMPRSS6	Sulfurtransferase	21.14
Q76LV2	HSP90AA1	Heat shock protein HSP 90-alpha	20.88
A0A452DJ66	TUBA1D	Tubulin alpha chain	20.81
Q3MHM5	TUBB4B	Tubulin beta-4B chain	20.77
A0A4W2GV41	CLTC	Clathrin heavy chain	20.45
A0A4W2CTC9	A2M	Uncharacterized protein	20.36
A0A4W2FJT1	HSPA8	Heat shock protein family A (Hsp70) member 8	20.32
A0A4W2CIB6	VIM	Vimentin	20.16
A0A4W2G1A4	KRT76	Keratin, type II cytoskeletal 2 oral-like	20.15
F1MU12	KRT8	Keratin, type II cytoskeletal 8	20.08
A0A4W2HN26	FLNA	Filamin A	19.98
F1MD77	LAMC1	Laminin subunit gamma 1	19.95
A0A4W2EG96	VCP	15S Mg(2+)-ATPase p97 subunit	19.85
E9RHW1	HSPB1	Heat shock 27 kDa protein	19.85
A0A4W2C164	─	Uncharacterized protein	19.83
F1MZ85	VCAN	Versican core protein	19.79
(**B**) Top 30 most abundant proteins in CM-EVs from EXPL + EMB			
A0A4W2FD79	ZNF638	Zinc finger protein 638	23.25
A0A4W2DYQ2	ACTB	Actin beta	22.91
A0A4W2HPP0	─	Uncharacterized protein	22.80
P62803	─	Histone H4	22.62
Q3ZC07	ACTC1	Actin, alpha cardiac muscle 1	22.47
A0A4W2HHA6	─	Histone H2B	22.34
E1BAU6	INPP5E	Inositol polyphosphate-5-phosphatase E	22.17
A0A3Q1MDT7	─	Histone H4	21.94
Q76LV2	HSP90AA1	Heat shock protein HSP 90-alpha	21.61
A0A4W2DVZ1	TUBB	Tubulin beta chain	21.50
P10096	GAPDH	Glyceraldehyde-3-phosphate dehydrogenase	21.30
A0A4W2H221	FGA	Fibrinogen alpha chain	21.15
F1MGU7	FGG	Fibrinogen gamma-B chain	21.14
F6S1Q0	KRT18	Keratin 18	21.10
A0A452DJ66	TUBA1D	Tubulin alpha chain	21.07
A0A4W2CGL9	SMC3	Chondroitin sulfate proteoglycan 6	21.01
A0A4W2F0F2	TMPRSS6	Sulfurtransferase	20.97
Q3MHM5	TUBB4B	Tubulin beta-4B chain	20.81
A0A452DIF5	H1-2	H1.2 linker histone, cluster member	20.74
A0A4W2GV41	CLTC	Clathrin heavy chain	20.70
A0A4W2BUC1	COPG1	Coatomer subunit gamma	20.47
A0A4W2FJT1	HSPA8	Heat shock protein family A (Hsp70) member 8	20.42
A7MBJ5	CAND1	Cullin-associated NEDD8-dissociated protein 1	20.38
F1MU12	KRT8	Keratin, type II cytoskeletal 8	20.32
A0A4W2CIB6	VIM	Vimentin	20.29
A0A4W2HN26	FLNA	Filamin A	20.20
A0A4W2CTC9	A2M	Uncharacterized protein	20.13
A0A4W2I521	EPHX2	Epoxide hydrolase 2	20.12
A0A4W2HR21	SPTAN1	Spectrin alpha, non-erythrocytic 1	20.07
F1MD77	LAMC1	Laminin subunit gamma 1	20.07
(**C**) Top 30 most abundant proteins in CM-EVs from EMB			
E1BAU6	INPP5E	Inositol polyphosphate-5-phosphatase E	23.98
A0A4W2FD79	ZNF638	Zinc finger protein 638	22.64
A0A4W2G1A4	KRT76	Keratin, type II cytoskeletal 2 oral-like	21.13
A0A4W2CGL9	SMC3	Chondroitin sulfate proteoglycan 6	20.73
A0A3Q1M4X6	SMUG1	Single-strand selective monofunctional uracil DNA glycosylase	20.00
F6S1Q0	KRT18	Keratin 18	19.12
A0A4W2ENX3	KRT75	Keratin 75	17.83
F1MF78	SYNE2	Spectrin repeat containing nuclear envelope protein 2	17.50
A0A4W2GNU2	ASNA1	ATPase ASNA1	17.38
A0A4W2D966	VPS13D	Vacuolar protein sorting 13 homolog D	17.04
A0A4W2H231	DSP	Desmoplakin	16.90
A0A4W2HA98	CEP135	Centrosomal protein 135	16.87
A0A4W2CM56	─	Aldo	16.85
F1MU12	KRT8	Keratin, type II cytoskeletal 8	16.79
A0A3Q1M1M7	JUP	Junction plakoglobin	16.68
A0A4W2DYQ2	ACTB	Actin beta	16.55
F1MIW8	DSG1	Desmoglein-1	16.54
A0A4W2E085	SRSF3	Serine and arginine rich splicing factor 3	16.46
A0A4W2BWI2	SEC16A	Protein transport protein sec16	16.42
A0A4W2GAK0	SYNE1	Spectrin repeat containing nuclear envelope protein 1	16.26
A0A4W2ECV0	AIDA	Axin interactor, dorsalization associated	16.25
Q0MRP5	LZ	Lysozyme	16.17
A0A4W2E9X9	SPATS2L	Uncharacterized protein	15.83
A0A4W2FQ19	UPK1B	Tetraspanin	15.80
A0A4W2GW83	ALB	Albumin	15.70
V6F7W7	UBE4A	Ubiquitin conjugation factor E4 A	15.60
A0A4W2H672	MELTF	Melanotransferrin	15.56
A0A4W2HUQ2	ATP5MF	ATP synthase membrane subunit f	15.39
P08728	KRT19	Keratin, type I cytoskeletal 19	15.33
A0A4W2IEJ4	MYOF	Myoferlin	15.29

*CM-EVs* Conditioned medium extracellular vesicles, *EXPL* Endometrial explants cultured alone, *EXPL+EMB* Endometrial explants co-cultured with blastocysts, *EMB* Blastocysts cultured alone

**Fig. 10:**
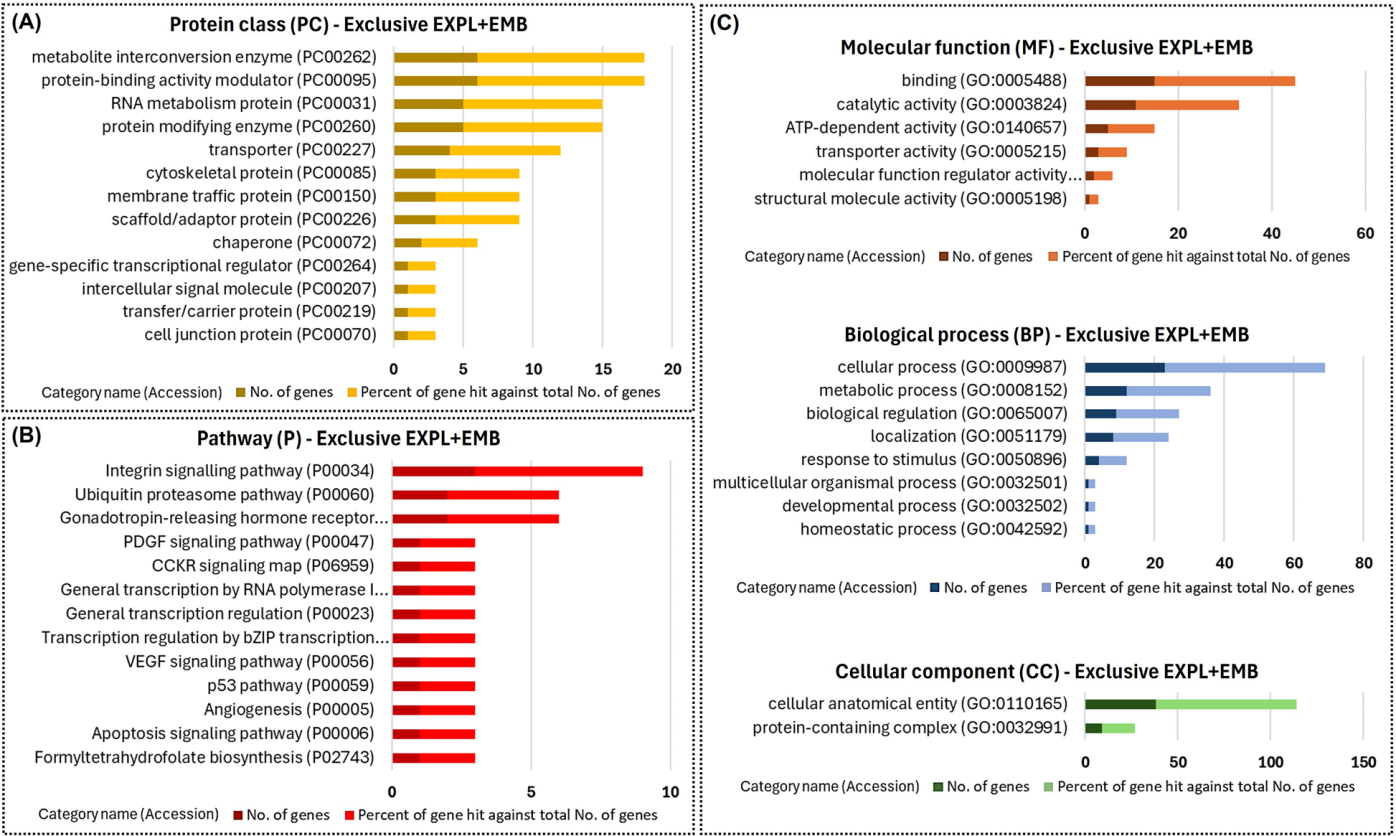
Functional enrichment of proteins exclusive to CM-EVs from EXPL + EMB. Fifty proteins were identified exclusively in the conditioned medium extracellular vesicles (CM-EVs) from endometrial explants co-cultured with blastocysts (EXPL + EMB). Protein class (**A**), pathways (**B**), and Gene Ontology (**C**) identified using the PANTHER 18.0 Classification System (https://pantherdb.org/). Darker bars indicate the number of genes associated with each category name, while lighter bars represent the percentage of these genes relative to the total number of genes in that category

Functional enrichment using the PANTHER database showed that the identified proteins were involved in different GO biological processes and pathways. The 50 proteins identified as being exclusively present in the EXPL + EMB group were mainly metabolite interconversion enzymes, protein modifying enzymes, RNA metabolism proteins, and metabolite interconversion enzymes (Fig. 10 A). These proteins participate in the cellular and metabolic process and biological regulation of pathways such as gonadotropin-releasing hormone receptors and integrin signaling (Fig. 10B and C).

In the quantitative analyses, we identified 33 differentially abundant proteins resulting from the synergistic effect between the embryo and blastocyst in vitro (Table 3). The quantitative analysis of the in vitro model was performed in pairs: EXPL vs. EMB, EMB vs. EXPL + EMB, and EXPL vs. EXPL + EMB. Once the differentially expressed proteins between these comparisons were identified, differentially abundant proteins not resulting from the embryo-maternal interaction (EXPL vs. EMB) were disregarded, leaving 33 differentially abundant proteins due to embryo-maternal interaction (Table 3).

**Table 3:** Differentially abundant proteins due to embryo–maternal interaction in vitro

Accession	Symbol	Description	EXPL vs. EMB (*P*-value)	EXPL vs. EXPL + EMB (*P*-value)	EMB vs. EXPL + EMB (*P*-value)	EXPL vs. EMB	EXPL vs. EXPL + EMB	EMB vs. EXPL + EMB
A0A4W2EGQ6	ARPC2	Arp2/3 complex 34 kDa subunit	0.0799	0.0435	0.0002	0	1	1
A0A4W2F3T9	LRRC17	Armadillo repeat containing 10	0.0773	0.0180	0.0002	0	1	1
A0A4W2F6C3	SURF4	Surfeit locus protein 4	0.2357	0.0371	0.0002	0	1	1
A5D7E8	PDIA3	Protein disulfide-isomerase	0.2348	0.0048	0.0003	0	1	1
Q9TU47	EIF6	Eukaryotic translation initiation factor 6	0.1417	0.0139	0.0003	0	1	1
A0A4W2GKH0	POLR2H	DNA-directed RNA polymerases I, II, and III subunit RPABC3	0.0631	0.0139	0.0003	0	1	1
Q3MHK9	FSCN1	Fascin	0.0594	0.0424	0.0004	0	1	1
A0A4W2DIA0	ERAP2	Aminopeptidase	0.2546	0.0224	0.0004	0	1	1
A0A4W2DCF6	PDCD10	Programmed cell death 10	0.1014	0.0262	0.0004	0	1	1
A0A4W2CF85	CAPNS1	Calcium-activated neutral proteinase small subunit	0.0512	0.0279	0.0005	0	1	1
A0A4W2EYS9	OSBPL9	Oxysterol-binding protein	0.1473	0.0094	0.0006	0	1	1
A5PKD6	GNB4	G protein subunit beta 4	0.3466	0.0030	0.0009	0	1	1
A0A4W2FR72	RAB25	RAB25, member RAS oncogene family	0.3466	0.0313	0.0011	0	1	1
A0A4W2HBQ0	YWHAH	Tyrosine 3-monooxygenase/tryptophan 5- monooxygenase activation protein eta	0.0545	0.0438	0.0022	0	1	1
A0A452DI24	GDI2	Rab GDP dissociation inhibitor	0.2967	0.0121	0.0032	0	1	1
V6F832	CRYAB	Alpha(B)-crystallin	0.2347	0.0374	0.0033	0	1	1
Q1RMW9	GOLPH3	Golgi phosphoprotein 3	0.1872	0.0433	0.0040	0	1	1
Q2T9M8	SNAP23	Synaptosomal-associated protein	0.1281	0.0364	0.0046	0	1	1
P37980	PPA1	Inorganic pyrophosphatase	0.3466	0.0179	0.0050	0	1	1
P07857	SCP2	Sterol carrier protein 2	0.0877	0.0395	0.0053	0	1	1
Q2KIW9	CMPK1	UMP-CMP kinase	0.3466	0.0103	0.0055	0	1	1
A0A4W2DWW9	ATE1	Arginyl-tRNA–protein transferase 1	0.1411	0.0310	0.0066	0	1	1
A0A4W2DB49	NAP1L1	Nucleosome assembly protein 1 Like 1	0.0536	0.0441	0.0068	0	1	1
A0A4W2HIZ5	FKBP9	Peptidylprolyl isomerase	0.3466	0.0357	0.0074	0	1	1
A8KC77	EIF5	eIF5 protein (Fragment)	0.3466	0.0211	0.0091	0	1	1
A0A4W2I2Z9	GSK3A	[Tau protein] kinase	0.3466	0.0392	0.0116	0	1	1
A0A4W2C0I1	─	Elongation factor 1-alpha	0.1449	0.0434	0.0119	0	1	1
P21856	GDI1	Rab GDP dissociation inhibitor alpha	0.3466	0.0332	0.0129	0	1	1
F1N1F9	SOX17	SRY-box transcription factor 17	0.2611	0.0318	0.0134	0	1	1
A0A4W2E056	FAT2	FAT atypical cadherin 2	0.3466	0.0323	0.0153	0	1	1
E1BB38	SRP72	Signal recognition particle subunit SRP72	0.0579	0.0326	0.0270	0	1	1
Q3SZX8	CCDC25	Coiled-coil domain-containing protein 25	0.3466	0.0496	0.0270	0	1	1
A0A4W2H076	STAG1	Stromal antigen 1	0.3466	0.0252	0.0340	0	1	1

*EXPL* Endometrial explants cultured alone, *EXPL* + *EMB* Endometrial explants co-cultured with blastocysts, *EMB* Blastocysts cultured alone. 0, No significant change, 1, Significant change (*P* < 0.05)

Thirty-three proteins were differentially abundant in conditioned medium extracellular vesicles (CM-EVs) resulting from the synergistic effect of embryo–maternal interaction in the in vitro model

**Fig. 11:**
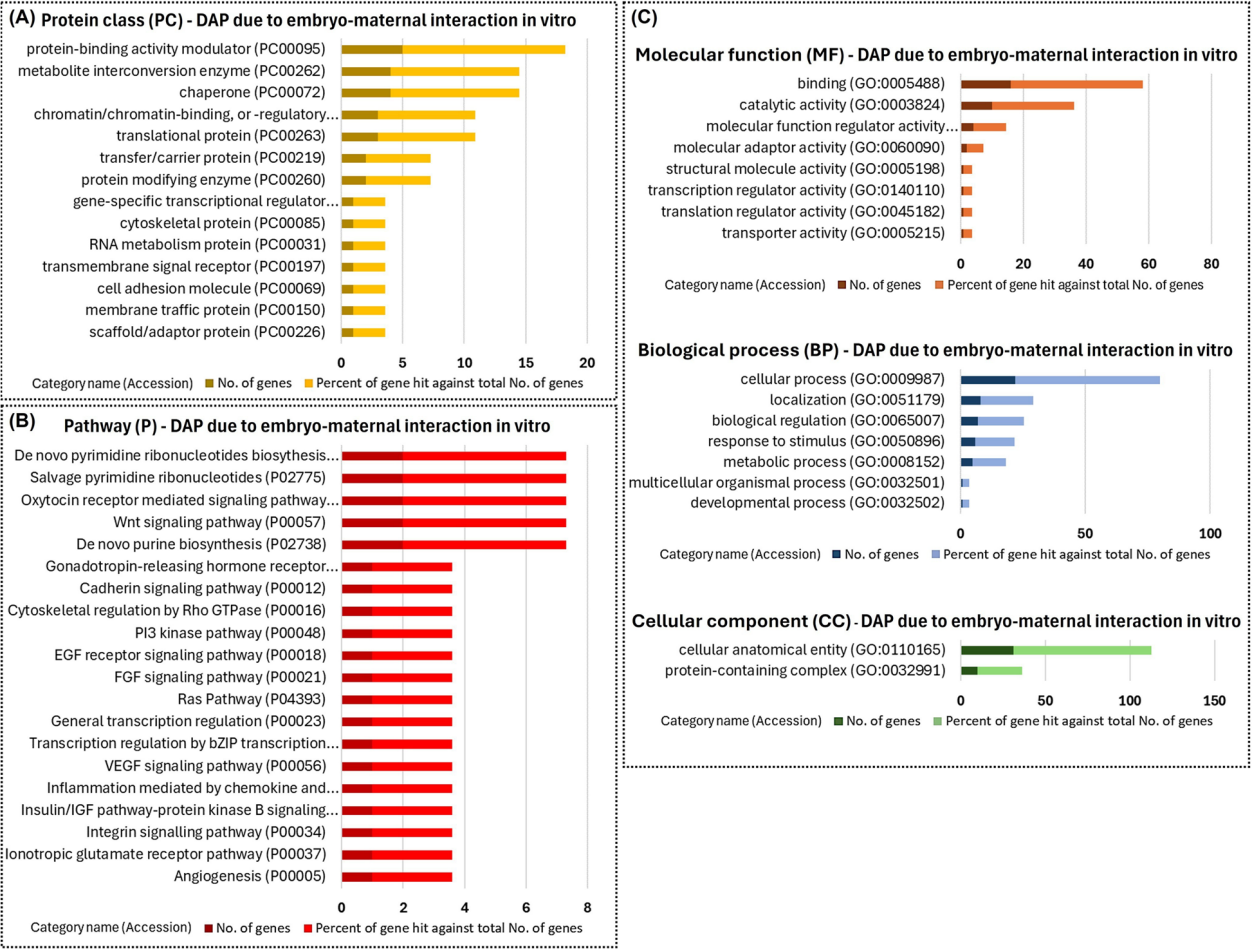
Functional enrichment of differentially abundant proteins due to embryo–maternal interaction in vitro. Thirty-three proteins were differentially abundant in conditioned medium extracellular vesicles (CM-EVs) resulting from the synergistic effect of embryo–maternal interaction in the in vitro model. Protein class (**A**), pathways (**B**), and Gene Ontology (**C**) identified using the PANTHER 18.0 Classification System (https://pantherdb.org/). Darker bars indicate the number of genes associated with each category name, while lighter bars represent the percentage of these genes relative to the total number of genes in that category

The PANTHER database was utilized to investigate the GO biological processes and pathways of these proteins. The proteins resulting from the synergistic effect between the embryo and blastocyst in vitro were primarily protein-binding modulators, metabolite interconversion enzymes, and chaperones (Fig. 11A). These proteins were associated with binding functions and processes such as biological regulation and response to stimuli (Fig. 11C). Additionally, they were involved in pathways such as Wnt signaling, PI3 kinase, and Ras (Fig. 11B).

#### Comparison of in vivo model and in vitro model

To investigate the distinctions and similarities in the embryo-maternal communication between in vivo and in vitro models, we conducted a comparative analysis using UF-EVs from CYCLIC heifers with CM-EVs from EXPL, and UF-EVs from PREGNANT heifers with CM-EVs from EXPL + EMB.

**Fig. 12:**
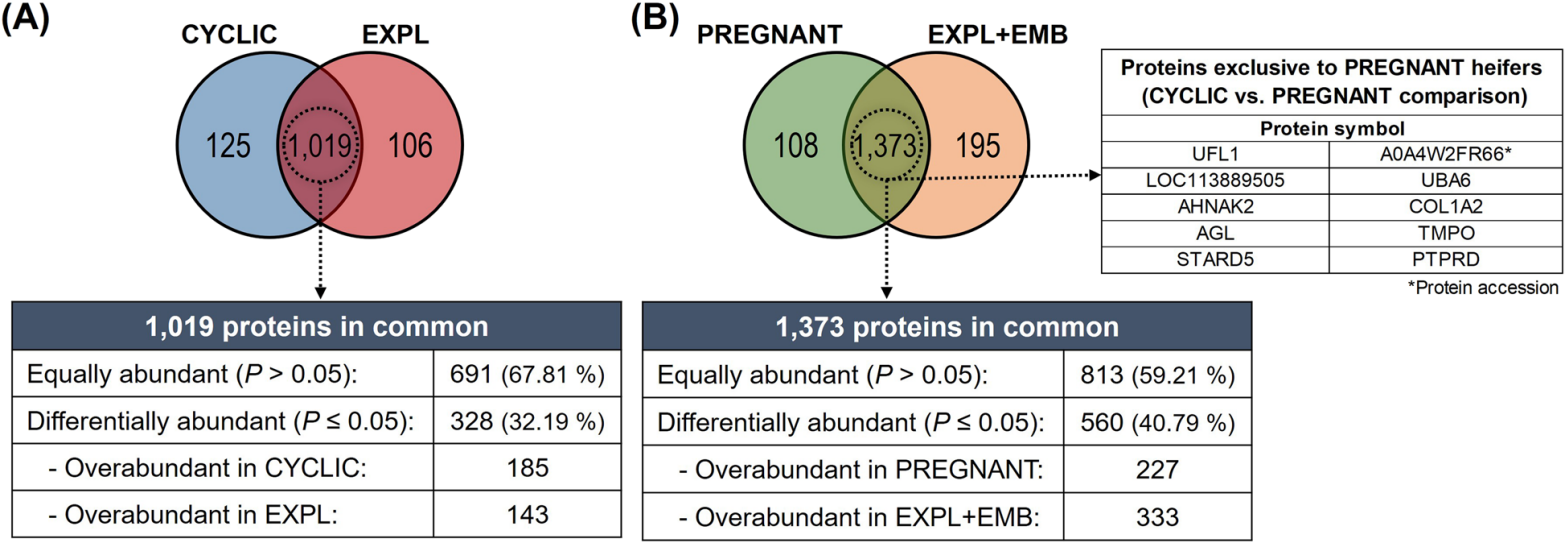
Comparison of protein identification between in vivo and in vitro models. **A** Venn diagram showing the proteins found in uterine fluid extracellular vesicles (UF-EVs) from CYCLIC heifers versus conditioned medium extracellular vesicles (CM-EVs) from endometrial explants cultured alone in vitro (EXPL). **B** Venn diagram showing the proteins in UF-EVs from PREGNANT heifers versus CM-EVs from endometrial explants cultured with blastocysts (EXPL + EMB) in vitro. Additionally, it identifies 10 proteins exclusively present in UF-EVs from PREGNANT heifers that were also detected in CM-EVs from EXPL + EMB. Proteins were considered ‘exclusive’ if detected in at least three out of five replicates within one group but absent in all samples of other groups

The analysis of the UF-EVs from CYCLIC heifers versus CM-EVs from EXPL (Fig. 12A) revealed 125 proteins exclusive to CYCLIC heifers, 106 unique to EXPL, and 1,019 proteins shared between them, among which 328 (32.2%) were identified as differentially abundant proteins. Similarly, in the comparison of UF-EVs from PREGNANT heifers with CM-EVs from EXPL + EMB (Fig. 12B), we identified 108 proteins exclusive to PREGNANT heifers, 195 unique to EXPL + EMB, and 1,373 proteins in common. Among these, 560 proteins (40.8%) were identified as differentially abundant proteins. Additionally, 10 proteins exclusively identified in PREGNANT when compared with CYCLIC heifers were also present in the EXPL + EMB group in vitro (Fig. 12B, right table).

Functional enrichment analysis using PANTHER revealed distinct GO biological processes when comparing proteins identified in UF-EVs from CYCLIC heifers and those in the CM-EVs from EXPL. Proteins exclusive to UF-EVs from CYCLIC heifers predominantly included metabolite interconversion enzymes, protein-binding activity modulators, membrane traffic proteins, and proteins modifying enzymes (Additional file 6A). Proteins unique to CM-EVs from EXPL were primarily metabolite interconversion enzymes, protein modifying enzymes, and RNA metabolism proteins (Additional file 6B). Equally abundant proteins consisted mainly of metabolite interconversion enzymes, protein modifying enzymes, and cytoskeletal proteins (Additional file 6C). Differentially abundant proteins were largely translational proteins, metabolite interconversion enzymes, and protein modifying enzymes (Additional file 6D). Both equally abundant and differentially abundant proteins contribute to binding, cellular, metabolic, and biological regulation, as well as to response to stimulus.

Proteins found exclusively in UF-EVs from PREGNANT heifers were mainly protein-binding activity modulators and metabolite interconversion enzymes (Additional file 7A). Proteins unique to CM-EVs from EXPL + EMB primarily included cytoskeletal proteins, protein modifying enzymes, and metabolite interconversion enzymes (Additional file 7B). Equally abundant proteins predominantly consisted of membrane traffic proteins, protein modifying enzymes, and metabolite interconversion enzymes (Additional file 7C). Differentially abundant proteins were largely protein-modifying enzymes, translational proteins, and metabolite interconversion enzymes (Additional file 7D). Both equally abundant and differentially abundant proteins contribute to the response to stimulus, localization, biological regulation, metabolic process, and cellular process.

**Fig. 13:**
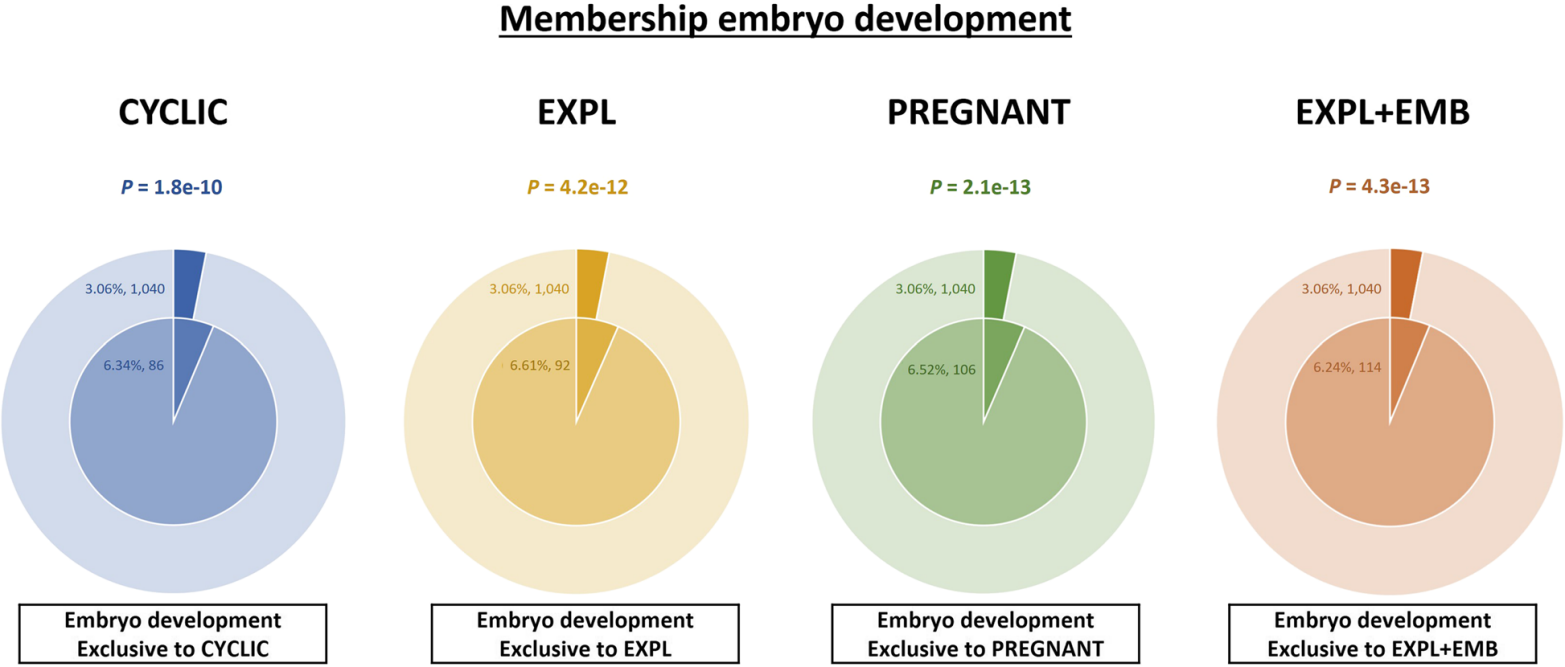
Functional membership analysis for proteins matching with “embryo development” term. Proteins identified in the uterine fluid extracellular vesicles from CYCLIC (blue pie chart) and PREGNANT heifers (green pie chart) and in the conditioned medium extracellular vesicles from explants cultured alone (EXPL; yellow pie chart) and explants co-cultured with embryos (EXPL + EMB; orange pie chart). The outer pie chart illustrates the count and proportion of proteins within the background dataset that were affiliated with the “embryo development” membership, whereas the inner pie chart presents the count and proportion of proteins in the specific input gene list associated with this membership. The *p*-value positioned above the pie charts indicates a statistically significant enrichment of the membership across all groups

Furthermore, functional membership analysis for proteins matching the term “embryo development” was conducted using proteins identified in both in vivo and in vitro models (Fig. 13). CM-EVs from EXPL + EMB had a higher number of proteins associated with embryo development (*n* = 114), followed by UF-EVs from PREGNANT heifers (*n* = 106). In UF-EVs from CYCLIC heifers, 86 proteins related to “embryo development” were identified, and 92 proteins related to embryo development were identified in the CM-EVs from EXPL. Additionally, the EXPL + EMB and PREGNANT groups showed lower *P*-values for this membership (4.3 × 10^–13^ and 2.1 × 10^–13^, respectively), indicating stronger statistical significance compared to CYCLIC (1.8 × 10^–10^) and EXPL (4.2 × 10^–12^).

## Discussion

To the best of our knowledge, this study is the first to characterize embryo-induced changes in the protein cargo of bovine uterine EVs on d 7 of pregnancy, conducted both in vivo and in vitro. These changes in protein content reflect embryo-maternal signaling through EVs and provide important insight into how these EVs may modulate key pathways involved in early pregnancy. In vivo, the blastocyst induces changes in UF-EV protein cargo related to inflammatory and immune responses, endometrial receptivity, and early embryonic development by promoting cell polarity, cell-cell adhesion, and stem cell differentiation. In vitro, embryo-induced alterations in CM-EVs include changes in proteins involved in embryonic development, regulation of stem cell differentiation, establishment and maintenance of cell polarity, IFNT-mediated cell signaling, endometrial receptivity, and immune modulation.

Moreover, EVs derived from the UF and CM exhibit distinct protein profiles, likely driven by different stimuli under in vitro and in vivo conditions, yet explants may offer a starting point for studying maternal-embryonic communication due to preserved characteristics among the models.

### Uterine and embryonic EVs

EVs have been recognized as constituents of the bovine UF [21]. In addition, embryonic EVs have been detected in spent culture media [50]. UF-EVs cargo, including miRNAs [21, 33] and proteins [16, 23, 32], have been investigated during the estrous cycle, with miRNA content changes also observed in the presence of multiple d 7 embryos [22]. Unlike prior studies, we identified the changes in the protein cargo of UF-EVs resulting from the presence of a single embryo at a more precise time-point by using synchronized heifers instead of abattoir-derived tissues. Moreover, although the protein cargo of UF-EVs has been analyzed in other ruminant species, such as sheep [18, 24] and goats [26], these studies focused on d 10 to 16, while we examined the changes occurring during the early pre-implantation period (d 7) of pregnancy.

In vitro, EVs from BEEC monolayer cultures have been shown to mediate embryo-maternal communication and enhance embryo development [51]; however, their cargo remains largely unexplored. Unlike previous studies, we employed endometrial explants to investigate embryo-maternal communication via EV protein content. Explants preserve both cellular and extracellular architecture, facilitating communication between different populations of endometrial cells. Studies have demonstrated that a 6-h incubation period is sufficient to induce significant changes in response to the embryo while preserving the structural and functional integrity of the explants [12, 39]. Additionally, previous studies have employed endometrial explants to investigate immunity and inflammation [36], IFNT modulation, and conceptus origin (in vivo vs. in vitro), sex [36], and size [52] effect on the endometrium.

Moreover, Passaro et al. [12] reported that d 8 embryos induced upregulation of ISG expression in endometrial explants, and that blastocyst-conditioned medium alone induces a response comparable to that seen with direct blastocyst-endometrium contact [12], suggesting that embryotrophins, including EVs, mediate this communication. EVs generated by in vitro-produced bovine blastocysts have been documented by several groups [53]. Most studies investigating the cargo of embryonic EV have focused on their miRNA profiles under different oxygen tensions [54], varying embryo viability [55], or developmental origin (in vitro vs. in vivo) [56, 57], with more recent attention given to their DNA content [58]. In contrast, we investigated the protein cargo of blastocyst-derived EVs in the context of embryo-maternal communication in vitro.

### In vivo model: embryo-induced alteration in UF-EVs

#### UF-EVs in early embryonic development

Extracellular matrix (ECM) components, cytoskeleton reorganization, and adhesion proteins are crucial for maintaining pluripotency and embryonic stem cell differentiation, thereby influencing blastocyst formation [59]. For example, trophoblast invasion and adhesion may depend on LAMB1, an ECM component overabundant in UF-EVs from PREGNANT heifers. Through interactions with other ECM components, LAMB1 uses a high-affinity receptor to interact with cells, promoting cell adhesion, motility, and organization throughout embryonic development [60]. In mice, LAMB1 knockout results in peri-implantation lethality around d 5.5 due to the failure of endoderm differentiation and impaired implantation [61]. Additionally, LAMB1 expression is elevated in human blastocyst trophectoderm during implantation [62] and in bovine conceptus during elongation [62]. These findings suggest that UF-EVs may modulate ECM components on d 7 blastocysts, potentially facilitating cell differentiation and blastocyst progression.

The CRB2 protein, exclusive to UF-EVs from PREGNANT heifers, is a critical member of the Crumbs cell polarity complex family, playing an essential role in early embryonic development. This transmembrane protein controls epithelial cell polarity and cell-cell adhesion, and its deficiency has been associated with embryonic lethality in mice [63]. The asymmetric localization of Crumbs in mouse blastomeres is essential for lineage development, preserving the polarity of epithelial cells and helping the polar signals to be transmitted at close junctions and the apical membrane [64]. Furthermore, the *CRB2* gene is upregulated in pig embryos [65], and single-cell gene expression studies of bovine blastocysts have revealed an overexpression of CRB2 in specific trophectoderm subpopulations [66], highlighting its importance in embryonic development and lineage specification. CRB2 also contributes to Hippo signaling, a regulator of cell fate determination and blastocyst formation [67]. The exclusive presence of CRB2 in UF-EVs from PREGNANT heifers suggests that it may represent a maternal response induced by the embryo’s presence, possibly influencing embryonic cell polarity, cell–cell adhesion, and lineage specification.

#### UF-EVs in inflammatory and immune response

Several proteins exclusively identified in UF-EVs from PREGNANT are predicted to participate in the immune response, notably UFL1**.** UFL1 maintains cell homeostasis and the inflammatory response by controlling nuclear factor-κB (NF-κB) signaling [68]. The overexpression of UFL1 in bovine mammary epithelial cells and bovine ovarian granulosa cells reduces inflammatory response and decreases cell damage [68]. This overexpression also inhibited the activation of the TLR4/NF-κB pathway and mitigated lipopolysaccharide-induced endoplasmic reticulum stress, apoptosis, autophagy, and oxidative stress [69, 70]. Conceptus presence also modulates UFL1 expression in the endometrium of pregnant swine, with significantly higher gene expression observed during allogeneic pregnancies (resulting from embryo transfer) compared to hemi-allogeneic pregnancies (resulting from artificial insemination) [71]. The exclusive presence of UFL1 in UF-EVs from PREGNANT heifers strongly suggests that these vesicles actively modulate endometrial function by delivering UFL1 and other regulatory proteins, shaping the local inflammatory response and enhancing endometrial receptivity to the embryo.

#### UF-EVs in endometrial receptivity

During the estrous cycle and pregnancy, the endometrium undergoes substantial functional alterations and remodeling necessary for successful implantation and placentation [72]. In our results, ECM-related proteins were modulated by the embryo presence and are associated with ECM organization, remodeling, and cell adhesion processes that could modulate uterine receptivity. COL1A2 is related to ECM organization within the pregnant endometrium [73], suggesting that the embryo could modulate ECM components, mainly during bovine maternal recognition of pregnancy (MRP). Indeed, COL1A2 facilitates bidirectional communication between the conceptus and endometrium in bovine during MRP [74]. Additionally, COL1A2 overexpression was observed in bovine endometrial epithelial cells treated with UF-EVs from d 17 to 20 of pregnancy [75].

Moreover, the ECM component LAMC1, involved in adhesion, migration, differentiation, and invasiveness, particularly in tumor cells [76], may also play a role in the endometrium. In mice, inhibition of Lamc1 negatively affects embryo implantation [77], while in ewes, LAMC1 expression increases in the endometrium on d 17 of pregnancy [78]. Similarly, in pregnant mares, LAMC1 shows higher relative expression in the endometrium on d 11 of pregnancy [79]. Although previous research indicated that changes in the distribution pattern of ECMs in the bovine endometrium are more evident by d 14 of pregnancy [72], our findings suggest that such modulation may initiate as early as d 7. Hence, in the embryo’s presence, COL1A2 and LAMC1 within UF-EVs may mediate endometrial ECM remodeling and assist endometrial receptivity.

#### UF-EVs as biomarkers of developmental competence

Proteins that may signal developmental competence were identified in UF-EVs from PREGNANT heifers. Heat shock proteins (HSPs) maintain intracellular homeostasis by regulating protein folding and exhibiting anti-apoptotic effects [80]. HSPA2, an HSPA (HSP70) family member, is also associated with cancer cell growth, survival, and male fertility, notably through its role in spermatid-specific chromatin remodeling during sperm cell differentiation and maturation [80]. Its deficiency is associated with male infertility and predicts failure in assisted reproductive technologies [80]. Although HSPA2 role beyond spermatogenesis is unclear, recent studies have identified HSPA2 in the bovine UF proteome during the periovulatory period of the estrous cycle [81], during pregnancy at d 10 and 13 [82], as well as in UF-EVs during peri-implantation periods (d 17, 20, and 22) [16] and in OF-EVs at d 3.5 of pregnancy [35].

Interestingly, higher gene expression of HSPA2 has been observed in bovine blastocysts compared to degenerate embryos [83], suggesting its involvement in embryo quality. Of note, other genes encoding proteins overabundant in UF-EVs from PREGNANT heifers (TTLL12 and PSMB2) have been associated with embryo quality and oocyte competence. Although their functions are not well characterized in reproduction, the *TTLL12* gene was found to be increased in a competent blastocyst that resulted in pregnancy [84], while the *PSMB2* gene has been linked to the competence of bovine oocytes [85]. These findings highlight the potential of UF-EV proteins as biomarkers of embryonic developmental competence; however, the specific roles of this protein in reproductive processes remain to be fully elucidated and warrant further investigation.

### In vitro model: embryo-induced alteration in CM-EVs

#### CM-EVs in early embryonic development

Embryo-induced alteration in CM-EVs may contribute to embryonic development by supporting stem cell differentiation and establishment and maintenance of cell polarity. Notably, NELFB is critical for cell proliferation and survival; its loss causes inner cell mass deficiency and embryonic lethality during mice implantation, while in porcine, reduced NELFB gene expression lowers blastocyst formation by impairing embryonic genome activation [86]. Similarly, disrupting THO proteins, such as THOC5, leads to early embryonic lethality in mice [87]. THOC5 is needed to export mRNAs encoding essential pluripotency factors (Nanog, Sox2, and Klf4), and its knockout affects stem cell maintenance, differentiation, and proliferation during mouse blastocyst development [88]. Thus, the delivery of these proteins through EVs to the embryo may enhance embryo progression.

Furthermore, CXADR and CLDN7, exclusive to the EXPL + EMB group, support tight junction integrity during early embryonic development, which is essential for maintaining cell polarity and fate specification, guiding morphogenesis through mechanotransduction and signaling networks [89]. CXADR knockdown impairs mice implantation capacity due to defective trophoblast development [90], while in porcine, CXADR knockdown embryos fail to reach the blastocyst stage, and those that do exhibit incomplete expansion [91]. Similarly, CLDN7 knockdown in porcine embryos also reduces blastocyst formation and the number of cells [92]. Furthermore, CLDN7 also contributes to cell–cell adhesion in murine uterine epithelial cells, where it localizes to the cell base and lateral plasma membrane [93]. These findings underscore the importance of these proteins in both embryonic morphogenesis and the maintenance of endometrial stability during early development.

#### CM-EVs in embryotrophin-mediated responses

In ruminants, IFNT, the most well-characterized embryotrophic factor secreted by trophoblastic cells, supports pregnancy establishment through its antiluteolytic effect and gene stimulation in the endometrium [94]. IFNT within EVs has been reported in ovine conceptus-derived EVs during d 15 and 17 of pregnancy [95] and in bovine UF-EVs on d 17, 20, and 22 of pregnancy [16], coinciding with the period around the peak of IFNT production [96]. We have not detected the presence of IFNT in UF-EVs in vivo, nor in CM-EVs in vitro, as early as d 7 of pregnancy. Nevertheless, previous studies indicated that the secretion of IFNT by bovine pre-hatching blastocysts, as well as EVs derived from d 5 to 7 embryos, induces the expression of ISGs in endometrial cells [9, 19].

Consistent with prior studies, we identified changes in CM-EVs exclusive to EXP + EMB likely associated with the IFNT effects. CM-EVs from the EXP + EMB group contain proteins encoded by the IFNT-dependent genes *SCRN1*, *TUBA1A*, *MLEC*, *SCP2*, and *ERAP2* [97], as well as *EIF2AK2*, *GNB4*, *PPA1*, which are both IFNT-dependent and conceptus-induced genes [36, 97]. EIF2AK2 regulates protein synthesis to mediate inflammatory responses [98] and may influence STAT1 transcription, a key IFN signaling factor [99]. Additionally, the *EIF2AK2* gene is overexpressed in bovine oviductal epithelial cells in the embryo’s presence [100], overabundant in pregnant ewes endometrium during the peri-implantation [101], but reduced in the endometrium of subfertile cows in early pregnancy [102], suggesting its role in proper endometrial function. Although further research is needed to elucidate the specific roles of these proteins in the endometrium, our findings strongly suggest that the presence of SCRN1, TUBA1A, MLEC, EIF2AK2, ERAP2, GNB4, PPA1, and SCP2 within EVs reflects active embryo-maternal communication mediated by embryotropins, potentially orchestrated through IFNT signaling pathways.

#### CM-EVs in endometrial receptivity and immune response

Several proteins exclusive to the EXPL + EMB group are implicated in endometrial receptivity. For example, LCN2 is mainly associated with tissue rearrangement and pregnancy in the female reproductive system [103]. In mares, LCN2 is upregulated in the endometrium at the site of conceptus implantation, likely driven by the conceptus, and related to endometrial innate immune responses [104]. In bovine, LCN2 levels in UF-EVs increase during the estrous cycle (d 0–16) and are also related to immune modulation [23]. Furthermore, LCN2 is upregulated in the bovine endometrium in response to pre-implantation factors [73], suggesting its modulation by the embryo-maternal communication. Therefore, LCN2 EV cargo may modulate endometrial receptivity by regulating inflammatory responses and facilitating embryo development in the uterus.

Moreover, Prostaglandin E Synthase 2 (PTGES2), exclusive to CM-EVs from EXP + EMB, may also play a significant role in uterine receptivity and conceptus development. Although our results did not detect PTGES2 in vivo within UF-EVs, the *PTGES2* gene is upregulated in the oviduct of pregnant cows [105] and is expressed in the bovine endometrium on d 7 of pregnancy [9]. PTGES2 catalyzes the synthesis of PGE2 from PGH2 in the endometrium [106]. Therefore, we propose that the delivery of PTGES2 via EVs may enhance these processes by facilitating PGE2 production in the bovine endometrium. Notably, PGE2 plays a critical role in supporting pre-implantation embryo development, luteotrophic signaling, and modulating endometrial receptivity and immune tolerance [107–110].

### In vivo vs. in vitro models

EVs derived from in vivo and in vitro conditions exhibit distinct qualitative and quantitative differences in their protein cargo. Endometrial explants were employed as an experimental model to approximate in vitro conditions as closely as possible to the in vivo uterine environment. In contrast to two-dimensional monocultures, explants preserve the native three-dimensional architecture and cellular heterogeneity of the endometrium [39], thereby offering a more physiologically relevant platform for investigating embryo-maternal interactions. Although this model presents inherent limitations, such as a restricted culture window and the number of embryos required per experimental condition, it effectively served our objective of capturing the early interaction between d 7 blastocysts and maternal tissue in vitro. As discussed above, both models show embryo-induced modifications in EV cargo that potentially support early embryonic development, endometrial receptivity, and immune response, yet these modifications involve different sets of proteins.

In the context of embryonic development, UF-EV cargo changes are associated with the regulation of embryonic cell polarity, cell–cell adhesion, and lineage specification through proteins like LAMB1 and CRB2. In contrast, changes in the in vitro model are associated with stem cell differentiation and establishment and maintenance of cell polarity through proteins including NELFB, THOC5, CXADR, and CLDN7. Moreover, changes in the EV protein cargo due to embryo-maternal communication facilitated by embryotrophins, likely via IFNT signaling, were detected only in CM-EVs, which may be attributed to signal amplification in the in vitro system due to the presence of multiple embryos, whereas in vivo, such changes might be less detectable at early stages of pregnancy. These differences in EV protein cargo, likely driven by distinct stimuli in in vitro and in vivo conditions, suggest that embryo-maternal communication employs unique molecular signals in each model while promoting similar developmental outcomes.

Despite these distinctions, it is noteworthy that 59.2% of proteins identified in both the PREGNANT and EXPL + EMB were equally abundant in both in vivo and in vitro models. These proteins are involved in pathways critical to embryo development, including Wnt (SFRP1, PPP2CA, GNB1, GNG2, PRKCI, GSK3B, GNB2, PPP2R5D, CSNK1A1, CSNK2A1, CDH1, and HDAC1), Ras (RPS6KA1, RPS6KA3, PAK2, RAC1, RHOA, GSK3B, CDC42, MAPK1, RAC1, STAT1, STAT3, and KRAS), and PI3K (GNAI1, GNB1, GNB2, YWHAZ, GSK3B, and KRAS) [111–113]. SFRP1 influences cell differentiation and growth during embryogenesis, ensuring proper embryonic modeling during early development [114]; while GSK3B and CSNK1A1 are related to proper cell differentiation [115]. Furthermore, *GSK3B* gene expression is positively correlated with successful embryo development [116]. In addition, HDAC1 regulates gene expression via epigenetic modifications, balancing cell proliferation and differentiation during embryonic development [117]. Similarly, STAT1 and STAT3 are involved in the regulation of cell proliferation and differentiation and are also involved in IFN-mediated cell signaling and immune responses [118]. These results highlight that essential signaling pathways for embryo development are present both in vivo and in vitro conditions.

On the other hand, 108 proteins were exclusive to UF-EVs from PREGNANT heifers compared to the EXPL + EMB group, including 11 (NF2, TSC1, SPINT2, BYSL, TFEB, ZNF358, TERF2, TULP3, VPS52, MARCKS, and CRB2) associated with embryo development, as indicated by Metascape tool. NF2 regulates the Hippo pathway during pre-implantation, controlling cell fate specification in mouse and bovine embryos [119], with its inhibition disrupting trophectoderm and inner cell mass segregation [120]. BYSL supports mice blastocyst formation by activation of the translational machinery [121]. Moreover, although its role remains unclear, *MARCKS* gene expression is associated with embryo competence [84]. Additionally, CRB2, related to cell polarity and cell–cell adhesion as previously discussed, was also exclusively detected in UF-EVs from PREGNANT heifers compared to CYCLIC ones. In this sense, EVs from in vivo conditions may offer greater benefits to embryonic development. Indeed, recent studies have indicated that EVs from the UF from cyclic cows and BEEC sources exert different effects on in vitro embryo development, with UF-EVs demonstrating superior effects on embryo quality [51]. Therefore, EVs derived from in vivo conditions, particularly UF-EVs from PREGNANT heifers, likely provide better support for embryonic development due to their distinct and specialized protein cargo. 

## Conclusion

In conclusion, this study characterized specific protein signatures of UF-EVs from PREGNANT heifers, which is likely due to the interactions established between the endometrium and the d 7 blastocyst. The blastocyst presence induces changes in UF-EVs protein cargo that are related to the regulation of inflammatory and immune responses, cell adhesion, polarity, and stem cell differentiation, potentially enhancing endometrial receptivity and supporting early embryonic development. Additionally, CM-EVs from endometrial explants cultured alone show different protein profiles compared to those co-cultured with d 7 blastocysts, as well as from the CM-EVs of d 7 blastocysts cultured alone. Embryo-induced alterations in CM-EVs are involved in embryonic development, regulation of stem cell differentiation, establishment and maintenance of cell polarity, IFNT-mediated cell signaling, endometrial receptivity, and immune modulation. Moreover, although uterine EVs from in vivo and in vitro sources exhibit distinct protein profiles, the preserved characteristics support the use of explants as a starting point for studying maternal-embryonic communication, with the acknowledgment that endometrium and embryos encounter unique in vitro conditions and stimuli compared to the maternal reproductive tract. Altogether, our findings reveal that the embryo-induced changes in the protein cargo of uterine EVs, observed in in vivo and in vitro models, position these EVs as key modulators of embryo-maternal communication, playing a pivotal role in supporting pre-implantation embryo development.

## Supplementary Information

Additional file 1. Experimental setup of the in vitro model. Extracellular vesicles (EVs) were isolated from the conditioned medium (CM) collected from three in vitro experimental groups: (1A–B) endometrial explants cultured alone (EXPL), (2A–B) endometrial explants co-cultured with blastocysts (EXPL + EMB), and (3A) blastocysts cultured alone (EMB).

Additional file 2. Strategy for quantitative analysis for in vitro comparison. Venn diagrams illustrate the identification of differentially abundant proteins (DAPs) between EXPL vs. EMB, EMB vs. EXPL + EMB, and EXPL vs. EXPL + EMB. The green circle represents DAPs in EXPL vs. EMB comparison.

Additional file 3. Negative controls for the characterization of extracellular vesicles (EVs) from uterine fluid (UF) and conditioned medium (CM). Phosphate‑buffered saline without calcium and magnesium (PBS^−/−^) was used as the negative control for both nanoparticle tracking analysis (NTA) and transmission electron microscopy (TEM). A. NTA of PBS^−/−^, showing 0 particles per frame. B. TEM images of PBS^−/−^, showing no vesicle-like structures.

Additional file 4. Proteomic profile of uterine fluid extracellular vesicles (UF-EVs). A. All 1459 proteins present in UF-EVs from CYCLIC heifers. B. All 1329 proteins present in UF-EVs from PREGNANT heifers. C. Differentially abundant proteins in UF-EVs from CYCLIC compared to PREGNANT heifers. D. Top 100 most abundant proteins in UF-EVs from CYCLIC and PREGNANT heifers.

Additional file 5. Proteomic profile of conditioned medium extracellular vesicles (CM-EVs). A. All 1501 proteins present in CM-EVs from explants cultured alone. B. All 1975 proteins present in CM-EVs from explants cocultured with embryos. C. All 82 proteins present in CM-EVs from embryos cultured alone. D. Top 100 most abundant proteins in CM-EVs from EXPL, EXPL+EMB and EMB.

Additional file 6. Functional enrichment of proteins identified in uterine fluid extracellular vesicles (UF-EVs) from CYCLIC heifers compared to conditioned medium extracellular vesicles (CM-EVs) from endometrial explants cultured alone (EXPL). A. Proteins (*n* =125) exclusive to UF-EVs from CYCLIC. B. Proteins (106) exclusive to CM-EVs form EXPL. C. Proteins (*n* = 691) equally abundant among CYCLIC vs. EXPL. D. Proteins (*n* = 328) differentially abundant among CYCLIC vs. EXPL.

Additional file 7. Functional enrichment of proteins identified in uterine fluid extracellular vesicles (UF-EVs) from PREGNANT heifers compared to conditioned medium extracellular vesicles (CM-EVs) from endometrial explants co-cultured with blastocysts (EXPL + EMB). A. Proteins (*n* =108) exclusive to UF-EVs from PREGNANT. B. Proteins (*n* = 195) exclusive to CM-EVs form EXPL + EMB. C. Eight hundred and thirteen proteins (*n* =813) equally abundant among PREGNANT vs. EXPL + EMB. D. Proteins (*n* = 560) differentially abundant among PREGNANT vs. EXPL + EMB.

## Data Availability

The mass spectrometry proteomics data have been deposited to the ProteomeXchange Consortium (https://proteomecentral.proteomexchange.org) via the iProX partner repository [122, 123] with the dataset identifier PXD053670.

## Abbreviations

BEEC: Bovine endometrial epithelial cells
CL: Corpus luteum
CM: Conditioned medium
CM-EVs: Conditioned medium extracellular vesicles
COCs: Cumulus-oocyte complexes
DAPs: Differentially abundant proteins
dFCS: EV-depleted fetal calf serum
EMB: Blastocysts cultured alone
EVs: Extracellular vesicles
EXPL: Endometrial explants cultured alone
EXPL + EMB: Endometrial explants co-cultured with blastocysts
FA: Formic acid
FC: Flow cytometry
FCS: Fetal calf serum
FDR: False discovery rate
FSC: Forward scatter
HSPs: Heat shock proteins
IFNT: Interferon tau
ISGs: Interferon-stimulated genes
IVC: In vitro culture
LFQ: Label-free quantification
miRNAs: MicroRNAs
MRP: Maternal recognition of pregnancy
NTA: Nanoparticle tracking analysis
PBS^−^^/^^−^: Phosphate-buffered saline without Ca^2+^ and Mg^2+^
PBS: Phosphate-buffered saline
PCA: Principal component analysis
PGF_2_α: Prostaglandin F_2_ alpha
PRID: Progesterone-releasing intravaginal device
PSM: Peptide-spectrum match
PTGES2: Prostaglandin E synthase 2
SEC: Size-exclusion chromatography
SOF: Synthetic oviduct fluid
SSC: Side scatter
TEM: Transmission electron microscopy
UF: Uterine fluid
UF-EVs: Uterine fluid extracellular vesicles
