# Supplementary figures and images for: Deciphering the dialogue between the bovine blastocyst and the uterus: embryo-induced alterations in extracellular vesicle protein content from an ex vivo model and the in vivo environment
Source: J Anim Sci Biotechnol. 2025 Oct 24;16:137. doi: 10.1186/s40104-025-01270-1 (PMC12551311; doi:10.1186/s40104-025-01270-1)

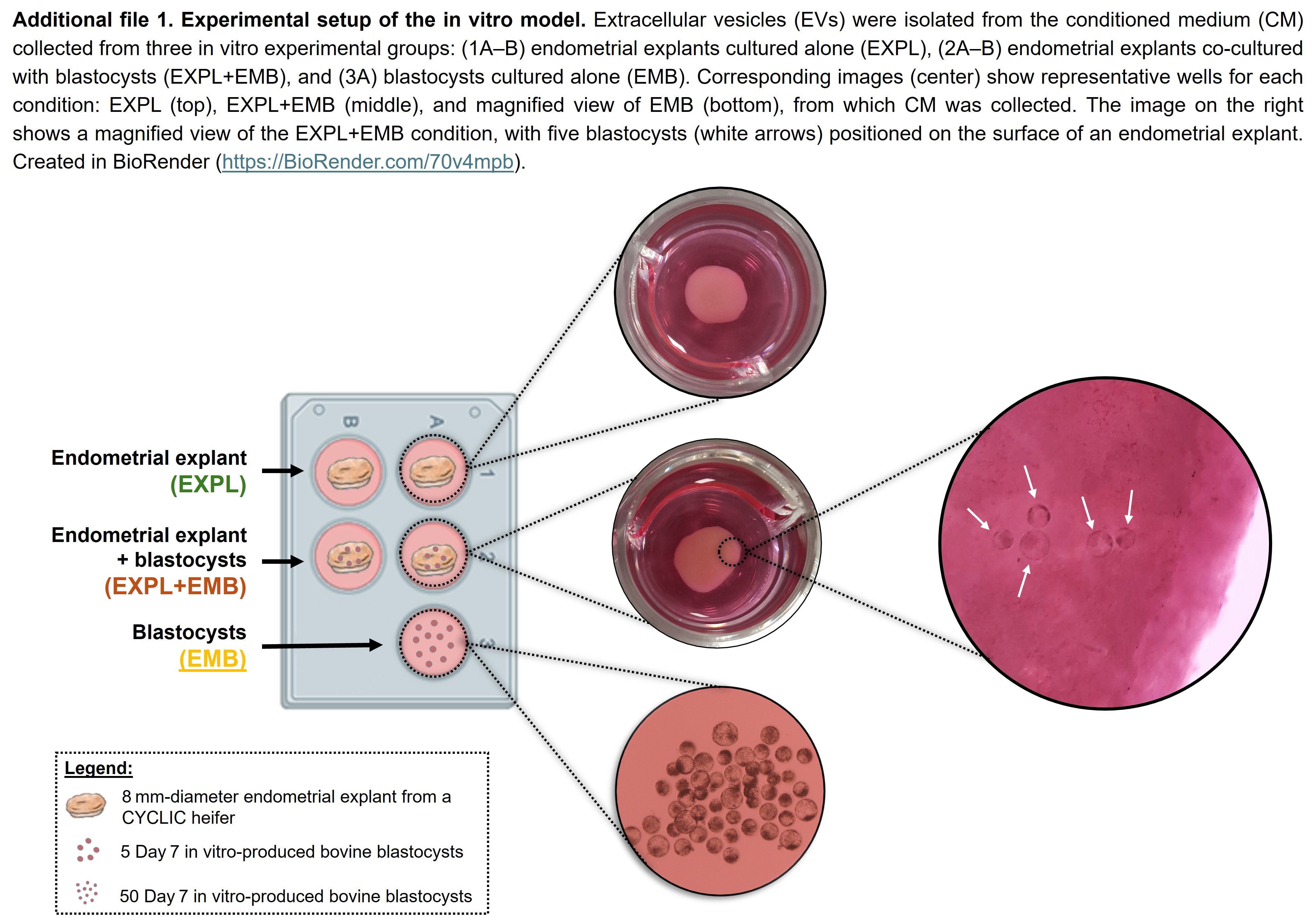

Supplement: Supplementary file 1 — Additional file 1. Experimental setup of the in vitro model. Extracellular vesicles (EVs) were isolated from the conditioned medium (CM) collected from three in vitro experimental groups: (1A–B) endometrial explants cultured alone (EXPL), (2A–B) endometrial explants co-cultured with blastocysts (EXPL + EMB), and (3A) blastocysts cultured alone (EMB). [file 40104_2025_1270_MOESM1_ESM.jpg]

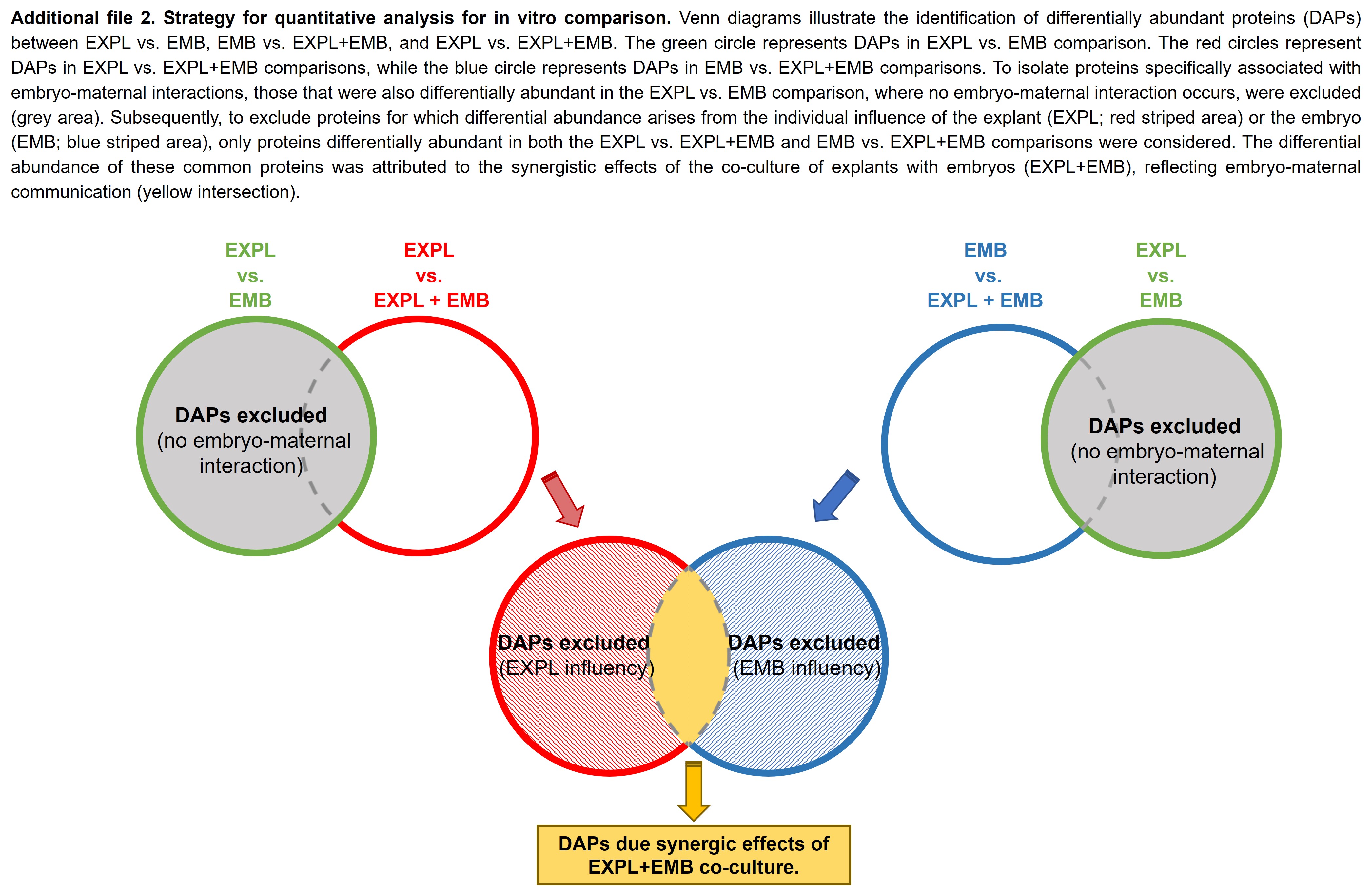

Supplement: Supplementary file 2 — Additional file 2. Strategy for quantitative analysis for in vitro comparison. Venn diagrams illustrate the identification of differentially abundant proteins (DAPs) between EXPL vs. EMB, EMB vs. EXPL + EMB, and EXPL vs. EXPL + EMB. The green circle represents DAPs in EXPL vs. EMB comparison. [file 40104_2025_1270_MOESM2_ESM.jpg]
