# Supplementary material for: Deciphering the dialogue between the bovine blastocyst and the uterus: embryo-induced alterations in extracellular vesicle protein content from an ex vivo model and the in vivo environment
Source: J Anim Sci Biotechnol. 2025 Oct 24;16:137. doi: 10.1186/s40104-025-01270-1 (PMC12551311; doi:10.1186/s40104-025-01270-1)

Additional File 3. Negative controls for the characterization of extracellular vesicles (EVs) from uterine fluid (UF) and conditioned medium (CM). Phosphate-buffered saline without calcium and magnesium ($PBS^{-/-}$) was used as the negative control for both nanoparticle tracking analysis (NTA) and transmission electron microscopy (TEM). (A) NTA of $PBS^{-/-}$, showing 0 particles per frame. (B) TEM images of $PBS^{-/-}$, showing no vesicle-like structures.

(A)

NANOSIGHT

PBS 2023-11-14 19-06-50

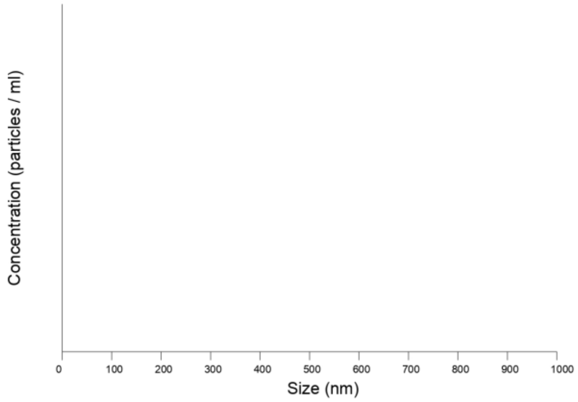

FTLA Concentration / Size graph for Experiment:
PBS 2023-11-14 19-06-50

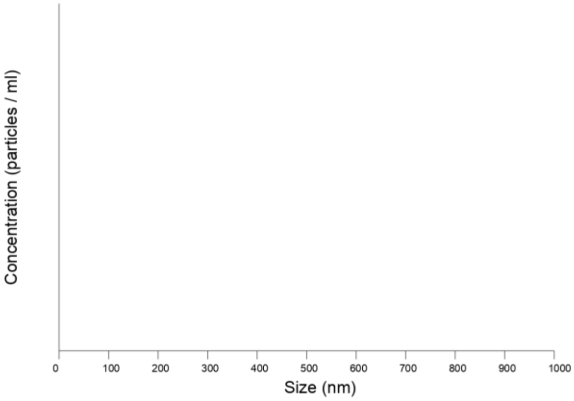

Averaged FTLA Concentration / Size for Experiment:
PBS 2023-11-14 19-06-50
Error bars indicate + / -1 standard error of the mean

Included Files

PBS 2023-11-14 19-07-06
PBS 2023-11-14 19-09-31

Details

NTA Version:	NTA 3.4 Build 3.4.4
Script Used:	SOP Standard Measurement 07-06-43PM 14~
Time Captured:	19:06:50 14/11/2023
Operator:	
Pre-treatment:	
Sample Name:	
Diluent:	
Remarks:	

Capture Settings

Camera Type:	CCD
Laser Type:	Red
Camera Level:	16
Slider Shutter:	1500
Slider Gain:	680
FPS	30.0
Number of Frames:	1800
Temperature:	21.1 ºC
Viscosity:	(Water) 1.0 cP
Dilution factor:	Dilution not recorded

Analysis Settings

Detect Threshold:	4
Blur Size:	Auto
Max Jump Distance:	Auto: 12.0 pix

Results

Stats: Merged Data

Mean:	0.0 nm
Mode:	0.0 nm
SD:	0.0 nm
D10:	0.0 nm
D50:	0.0 nm
D90:	0.0 nm

Stats: Mean +/- Standard Error

Mean:	0.0 +/- 0.0 nm
Mode:	0.0 +/- 0.0 nm
SD:	0.0 +/- 0.0 nm
D10:	0.0 +/- 0.0 nm
D50:	0.0 +/- 0.0 nm
D90:	0.0 +/- 0.0 nm
Concentration:	5.08e+04 +/- 3.05e+04 particles/ml
	0.0 +/- 0.0 particles/frame
	0.5 +/- 0.0 centres/frame

(B)

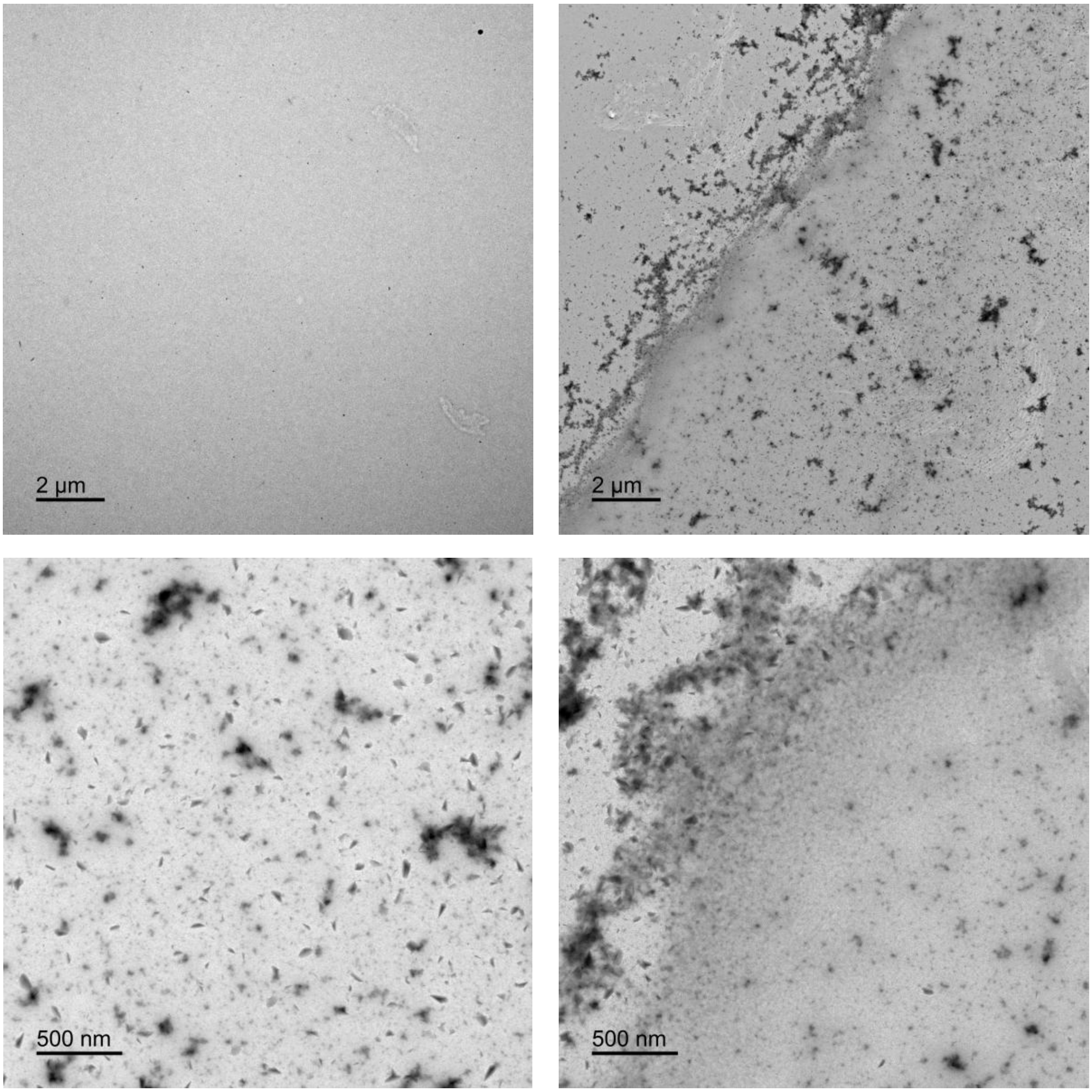

Supplement: Supplementary file 3 — Additional file 3. Negative controls for the characterization of extracellular vesicles (EVs) from uterine fluid (UF) and conditioned medium (CM). Phosphate‑buffered saline without calcium and magnesium (PBS−/−) was used as the negative control for both nanoparticle tracking analysis (NTA) and transmission electron microscopy (TEM). A. NTA of PBS−/−, showing 0 particles per frame. B. TEM images of PBS−/−, showing no vesicle-like structures. [file 40104_2025_1270_MOESM3_ESM.pdf]
